# Endogenous Oligodendroglial Alpha-Synuclein and TPPP/p25α Orchestrate Alpha-Synuclein Pathology in Experimental Multiple System Atrophy Models

**DOI:** 10.1007/s00401-019-02014-y

**Published:** 2019-04-22

**Authors:** Panagiota Mavroeidi, Fedra Arvanitaki, Anastasia-Kiriaki Karakitsou, Maria Vetsi, Ismini Kloukina, Markus Zweckstetter, Karin Giller, Stefan Becker, Zachary A. Sorrentino, Benoit I. Giasson, Poul Henning Jensen, Leonidas Stefanis, Maria Xilouri

**Affiliations:** 1 Center of Clinical Research, Experimental Surgery and Translational Research, Biomedical Research Foundation of the Academy of Athens, Greece; 2 Center of Basic Research, Biomedical Research Foundation of the Academy of Athens, Greece; 3 German Center for Neurodegenerative Diseases (DZNE), Von-Siebold-Str. 3a, 37075 Göttingen, Germany; 4 Department for NMR-based Structural Biology, Max Planck Institute for Biophysical Chemistry, Am Faßberg 11, 37077 Göttingen, Germany.; 5 Department of Neuroscience, University of Florida, Gainesville, FL 32610, USA.; 6 Center for Translational Research in Neurodegenerative Disease, University of Florida, Gainesville, FL 32610, USA; 7 McKnight Brain Institute, University of Florida, Gainesville, FL 32610, USA; 8 DANDRITE-Danish Research Institute of Translational Neuroscience & Department of Biomedicine, University of Aarhus, Denmark; 9 1st Department of Neurology, Eginition Hospital, National and Kapodistrian University of Athens, Medical School, Greece

**Keywords:** alpha-synuclein, multiple system atrophy, myelin, oligodendrocytes, seeding, tubulin polymerization promoting protein

## Abstract

Multiple system atrophy (MSA) is characterized by the presence of distinctive glial cytoplasmic inclusions (GCIs) within oligodendrocytes that contain the neuronal protein alpha-synuclein (aSyn) and the oligodendroglia-specific phosphoprotein TPPP/p25α. However, the role of oligodendroglial aSyn and p25α in the formation of aSyn-rich GCIs remains unclear. To address this conundrum, we have applied human aSyn (haSyn) pre-formed fibrils (PFFs) to rat wild-type (WT)-, haSyn-, or p25α-overexpressing oligodendroglial cells and to primary differentiated oligodendrocytes derived from WT, knockout (KO)-aSyn, and PLP-haSyn-transgenic mice. HaSyn PFFs are readily taken up by oligodendroglial cells and can recruit minute amounts of endogenous aSyn into the formation of insoluble, highly aggregated, pathological assemblies. The overexpression of haSyn or p25α accelerates the recruitment of endogenous protein and the generation of such aberrant species. In haSyn PFF-treated primary oligodendrocytes, the microtubule and myelin networks are disrupted, thus recapitulating a pathological hallmark of MSA, in a manner totally dependent upon the seeding of endogenous aSyn. Furthermore, using oligodendroglial and primary cortical cultures, we demonstrated that pathology-related S129 aSyn phosphorylation depends on aSyn and p25α protein load and may involve different aSyn “strains” present in oligodendroglial and neuronal synucleinopathies. Importantly, this hypothesis was further supported by data obtained from human post-mortem brain material derived from patients with MSA and dementia with Lewy bodies. Finally, delivery of haSyn PFFs into the mouse brain led to the formation of aberrant aSyn forms, including the endogenous protein, within oligodendroglia and evoked myelin decompaction in WT mice, but not in KO-aSyn mice. This line of research highlights the role of endogenous aSyn and p25α in the formation of pathological aSyn assemblies in oligodendrocytes and provides *in vivo* evidence of the contribution of oligodendroglial aSyn in the establishment of aSyn pathology in MSA.

## Introduction

Multiple system atrophy (MSA) is a fatal, adult-onset, sporadic neurodegenerative disorder of uncertain etiology with no effective treatment [14]. Neuropathologically, MSA is characterized by the accumulation of the neuronal presynaptic protein alpha-synuclein (aSyn) within oligodendrocytes, the myelin-producing cells of the central nervous system, forming glial cytoplasmic inclusions (GCIs), which represent the main histopathological hallmark of MSA [67, 73]. The origin of aSyn in oligodendrocytes of MSA patients is ambiguous, since it has been proposed that mature oligodendrocytes do not normally express this protein [9]. Some reports have shown the expression of *SNCA* (the gene encoding aSyn) mRNA in oligodendrocytes, suggesting the presence of the endogenous oligodendrocytic protein [9, 21, 56]. However, other studies have failed to detect oligodendroglial *SNCA* mRNA expression in the brains of MSA patients [35, 60], or in studies where *SNCA* mRNA was detected, no differences were observed between controls and MSA patients [3, 19]. Moreover, *in vitro* [13, 21, 23, 24, 48, 49] and *in vivo* studies [55, 58] demonstrated that exogenous recombinant or neuronally derived aSyn can be taken up by oligodendroglial cell lines, suggesting the neuron-to-glia transfer of aSyn. Recent evidence suggests that the “prion-like” transmission of misfolded aSyn may contribute to MSA disease risk [76]. Yet, little is known regarding the mechanisms underlying the selective transmission of aSyn pathology in oligodendrocytes of MSA brains.

Beyond aSyn, the oligodendroglial-specific phosphoprotein p25α (tubulin polymerization promoting protein, TPPP) is a major component of GCIs and facilitates aSyn aggregation *in vitro* [18, 25, 28, 61]. Under physiological conditions, p25α interacts with tubulin and myelin basic protein (MBP), thereby facilitating myelination [61]. In MSA, p25α is redistributed from the myelin sheath to the abnormally expanded oligodendroglial cell bodies, an event followed by a reduction of total MBP levels, myelin fragmentation, and accumulation of abnormal aSyn [61]. However, the role of p25α in the transmission and seeding of aSyn pathology has not been addressed.

In the present study, by utilizing pre-formed fibrils (PFFs) of human recombinant aSyn as seeds of pathological aSyn, we show that endogenous oligodendroglial aSyn, which is almost undetectable at baseline, is a major component of the misfolded aSyn assemblies formed in immortalized oligodendroglial cell lines and primary oligodendroglial cultures. More importantly, we demonstrate that endogenous aSyn plays a central role in the impairment of the myelin network in the living mouse brain, further highlighting the contribution of endogenous aSyn to the establishment of the pathology present in MSA brains. Finally, our study supports a central role for p25α in the seeding of misfolded aSyn and in the formation of pathological aSyn conformations, which are considered crucial events underlying the pathology observed in MSA brains.

## Methods

### Cell culture and treatments

The stable cell line OLN-p25α was established essentially as the OLN-AS7 line [51] by transfecting OLN-93 oligodendroglial cells with a pcDNA3.1 zeo(−)human p25α vector. The parental immortalized OLN-93 cell line (control cells) was derived from primary Wistar rat brain glial cultures [57]. All cells were grown in Dulbecco’s modified Eagle’s medium (D6429; Gibco, Invitrogen, Carlsbad, CA, USA) supplemented with 10% fetal bovine serum (10270; Gibco, Invitrogen, Carlsbad, CA, USA), 50 U/mL penicillin, and 50 μg/mL streptomycin. OLN-AS7 and OLN-p25α cells were maintained in 50 μg/mL Zeocin (R25001; Thermo Fisher Scientific, Waltham, MA, USA). The pcDNA3.1 zeo(−) plasmid expressing human p25α was generated as described previously [68]. Primary oligodendroglial progenitor cells derived from mixed glial cultures were prepared from P0-P3 neonatal knockout (KO)-aSyn, wild-type (WT)-aSyn or PLP-human aSyn (haSyn) transgenic mice, as described previously [32], and cultured in SATO medium [5]. Cultures of rat (embryonic day 18) cortical neurons were prepared as described previously [78] and maintained in Neurobasal medium (Invitrogen, Carlsbad, CA, USA), supplemented with B27 supplement (Invitrogen, Carlsbad, CA, USA), L-glutamine (0.5 mM), and penicillin/streptomycin (1%). A detailed description of the conditions utilized for plating the different cell types, transfections, and treatments are provided in Suppl. Methods (Online Resource 16).

### Preparation of haSyn PFFs

HaSyn fibrils were generated as described [6, 22], with several modifications. Shortly, before aggregation, monomeric aSyn in 50 mM HEPES (pH 7.4,) 100 mM NaCl, and 0.02% NaN_3_ was centrifuged for 1 h at 84.000 x *g*. The supernatant was ultra-filtrated (0.2 μm) and adjusted to 0.27 mM. Aggregation was performed for 12 days at 37°C. Fibrils were collected by ultracentrifugation, washed twice with phosphate-buffered saline (PBS), pH 7.3, at room temperature, and quantified by subtracting the amount of monomeric aSyn protein in the supernatant from the total protein used for aggregation. PFFs were resuspended in PBS (pH 7.3) at a final concentration of 4.5 mg/mL and a working stock solution was prepared at 1 mg/mL. The cells were incubated with 0.5–3 μg non-sonicated haSyn PFFs (as described above) or PBS as a control for the indicated time points and were either processed for confocal microscopy or lysed and collected for western blot analysis as described below.

### Subcellular fractionation and western immunoblotting

OLN cells incubated with haSyn PFFs for various time points were washed twice in ice-cold PBS and sequentially fractionated using buffers with increasing extraction strength, as described in Suppl. Methods (Online Resource 16). Primary oligodendroglial cells were lysed using RIPA buffer (150 mM NaCl, 25 mM Tris pH 7.6, 0.5% sodium deoxycholate, 0.1% SDS, 1% NP-40), left on ice for ~30 min, and centrifuged at 10,000 x *g* for 15 min at 4°C. Western blot analysis of protein extracts was performed as described previously [70] and the antibodies used are shown in Suppl. Table 2 (Online Resource 11). The intensity of each immunoreactive band was estimated by densitometric quantification using ImageJ software.

### Immunocytochemistry and confocal microscopy

OLN cell lines and primary mouse oligodendrocyte cultures cultured on poly-D-lysine-coated glass coverslips were treated with haSyn PFFs at the indicated time points. The cells were fixed with 4% paraformaldehyde for 40 min, blocked in 10% normal goat serum containing 0.4% Triton X-100 for 1 h at room temperature, and incubated with primary antibodies overnight at 4°C. The primary and fluorescent secondary antibodies used are shown in Suppl. Table 2 (Online Resource 11). Images were obtained using a Leica TCS SP5 confocal microscope combined with a dual (tandem) scanner. All confocal images were obtained under equivalent conditions of laser power, pinhole size, gain, and offset settings between the groups. For the transient transfections, quantifications were performed with the Imaris software suite (v7.7.2, Bitplane AG, Zurich, Switzerland), using a static set of parameters to isolate either GFP^+^ or p25α^+^ cell profiles, and then masking the channel containing the human aSyn (LB509 antibody), oxidized/nitrated aSyn (SYN-303 antibody) or the rodent aSyn (D37A6 antibody) signal using said profiles. The masking of the GFP^+^ or p25α^+^ signal was based on fluorescent intensity. ImageJ (v2.0.0) software was used to quantify relative protein levels expressed as mean fluorescence intensity or % area coverage, normalized to the total number of cells/field (the number of DAPI-stained nuclei).

### Transmission electron microscopy (EM)

#### Negative staining:

Specimens were prepared by adsorbing 5-μLdrops of fibrils (pre-and post-sonication) onto 200 mesh formvar-carbon film-bearing grids (Electron Microscopy Sciences, Hatfield, PA, USA), rinsing with water, and staining with a 2% w/w aqueous uranyl acetate solution for 2 min.

#### Preparation of cultured cells for EM and immuno-EM:

For conventional EM, the cells were fixed for 1 h at 37°C in 2.5% glutaraldehyde made up in 0.1 M phosphate buffer (pH 7.4) and processed for EM and immuno-EM as described in Supp. Methods (Online Resource 16).

#### EM for myelin integrity:

At 1 month post-injection, haSyn PFF-treated WT and KO-aSyn mice (n = 4/genotype) were perfused transcardially with 0.1M PBS (pH 7.2) at 37°C and then with 4% paraformaldehyde/1% glutaraldehyde. The brain was removed and the ipsilateral striatum was processed for EM analysis as described in Supp. Methods (Online Resource 16). In all EM procedures, the grids were examined in a Philips 420 transmission electron microscope at an acceleration voltage of 60 kV and photographed with a Megaview G2 CCD camera (Olympus SIS, Münster, Germany) and iTEM image capture software. In order to assess myelin integrity in the PFF-injected mice striatum, we quantified the g-ratio (ratio of inner axonal diameter to total outer diameter) and the % of axons with decompacted myelin [43]. For these quantifications, at least 100 randomly selected myelin sheaths that were cross-sectioned completely without artifacts and could be classified without doubt were counted. Semi-automated analysis of randomly selected myelin sheaths was carried out using a plug-in for ImageJ software as previously described [7].

### RNA extraction, cDNA synthesis and real-time PCR

Total RNA was extracted from OLN-93 cells treated with haSyn PFFs for 2, 12, and 48 h (or PBS) using TRIzol® (Ambion, Thermo Fisher Scientific, Waltham, MA, USA). Following digestion with 1 U/μg DNase I (Promega, Madison, WI, USA), 1 μg total RNA was used for first strand cDNA synthesis with the Moloney murine leukemia virus reverse transcription system (Promega, Madison, WI, USA) and utilized for real-time PCR, according to the manufacturer’s instructions, as described in Suppl. Methods (Online Resource 16).

### Autopsy case material

Human brain tissue was obtained through the University of Florida Neuromedicine Human Brain Tissue Bank according to protocols approved by the Institutional Review Board. Post-mortem pathological diagnoses were made according to standard neuropathological criteria [33, 66]. Cerebellar samples containing GCIs from three MSA cases were studied including the two major pathological subtypes: striatonigral degeneration and olivopontocerebellar atrophy (Online Resource 10). The cingulate cortex and midbrain containing cortical and brainstem Lewy Bodies (LBs), respectively, from three dementia with Lewy bodies (DLB) cases with concurrent Alzheimer’s disease pathology were also utilized.

### Immunohistochemistry and semi-quantitative scoring

Immunostaining of the sections was performed using standard methods [11]. The sections were rehydrated and subsequent antigen retrieval was performed in a steam bath for 30 min in a 0.2% Tween-20 solution for all antibodies utilized. Non-specific antibody binding was minimized with a 2% fetal bovine serum/0.1M Tris blocking solution; primary antibodies were diluted in the blocking solution and applied to tissue sections at 4°C overnight. Biotinylated secondary antibodies (Vector Laboratories, Burlingame, CA, USA) were diluted in the blocking solution and applied to the sections for 1 h at room temperature. An avidin-biotin complex system (VECTASTAIN ABC Elite Kit; Vector Laboratories, Burlingame, CA, USA) was used to enhance detection of the immunocomplexes, which were visualized using the chromogen 3,3′-diaminobenzidine (DAB Kit; KPL, Gaithersburg, MD, USA). For comparison, all MSA and DLB sections were exposed to DAB for the same amount of time for a given antibody. Tissue sections were counterstained with hematoxylin. All slides were digitally scanned using an Aperio ScanScope CS instrument (40× magnification; Aperio Technologies Inc., Vista, CA, USA), and images of representative areas of pathology were captured using ImageScope software (40× magnification; Aperio Technologies, Inc., Vista, CA, USA). Inclusion pathology was observed and assessed qualitatively and confirmed independently by a second observer.

### Animals

Eight-week-old male WT C57BL6/C3H (WT-aSyn), C57BL6/JOlaHsd aSyn null (KO-aSyn) (purchased from Harlan Laboratories), or C57BL6 PLP-haSyn transgenic (kindly provided by Dr. Nadia Stefanova, Innsbruck University) mice were housed (6–8 animals/cage) with free access to food and water under a 12-h light/dark cycle. All experimental procedures were approved by the Ethics Committee for the Use of Laboratory Animals in the Biomedical Research Foundation of Athens.

### Surgical procedures

All surgical procedures were performed under general isoflurane (B506; Abbott, Chicago, USA) anesthesia. Eight-week-old male WT or KO aSyn mice were placed in a stereotaxic frame (Kopf Instruments, Tujunga, CA, USA) and the right dorsal striatum was targeted using the following coordinates from the bregma: +0.5 mm anteroposterior, −1.6 mm mediolateral, and −3.4 mm dorsoventral according to the Mouse Brain Atlas [80]. A total of 2 μL haSyn PFF solution (final concentration, 2 μg/μL) or PBS was injected unilaterally at a constant flow rate of 0.1 μL/15 s using a pulled glass capillary (diameter, 60–80 mm) attached to a Hamilton syringe with a 22 gauge needle. Following delivery of haSyn PFFs or PBS, the capillary was held in place for 5 min and removed slowly from the brain.

### Immunohistochemistry and confocal microscopy

At 1 month post-injection, the animals were anaesthetized with isoflurane followed by intracardial perfusion with ice-cold PBS and then ice cold 4% paraformaldehyde solution (n = 4–5 animals/condition/genotype). The brains were removed and post-fixed for 24 h in the same preparation of paraformaldehyde and transferred to 15% sucrose for 24 h and finally in 30% sucrose until sectioning. The brains were sectioned through the coronal plane at 30 μm increments, and every section throughout the striatum and ventral midbrain was collected. Immunohistochemical staining was carried out in free-floating sections as described previously [79] using the primary and secondary antibodies described in the Immunocytochemistry and Confocal Microscopy section.

### Statistical analysis

The data are shown as the mean ± standard error (SE) of 3 or 5 independent experiments with triplicate samples/condition within each experiment. The mean values ± standard deviation (SD) of each experiment are also provided in Suppl. Table 3 (Online Resource 12). Image acquisition and analysis were performed by two independent examiners blinded to the true identity of the samples. Statistical analysis was carried out with GraphPad Prism 5 using one-way analysis of variance (ANOVA) with Tukey’s post-hoc test or two-way ANOVA with Bonferroni’s correction. Differences were considered significant for p < 0.05.

## Results

### Internalization of haSyn PFFs by oligodendroglial cell lines leads to the formation of Triton-insoluble aSyn species

In order to assess the uptake and seeding capacity of haSyn PFFs in oligodendroglial cells, we initially utilized the immortalized rat OLN-93 cell line, which expresses very low to non-detectable levels of endogenous aSyn and p25α, and the stable cell lines OLN-AS7 and OLN-p25α, which overexpress human WT-aSyn and p25α, respectively (Online Resource 1a). Stable or transient overexpression of haSyn and p25α in OLN cells was not accompanied by changes in cell viability (Online Resource 1b).

**Fig. 1:**
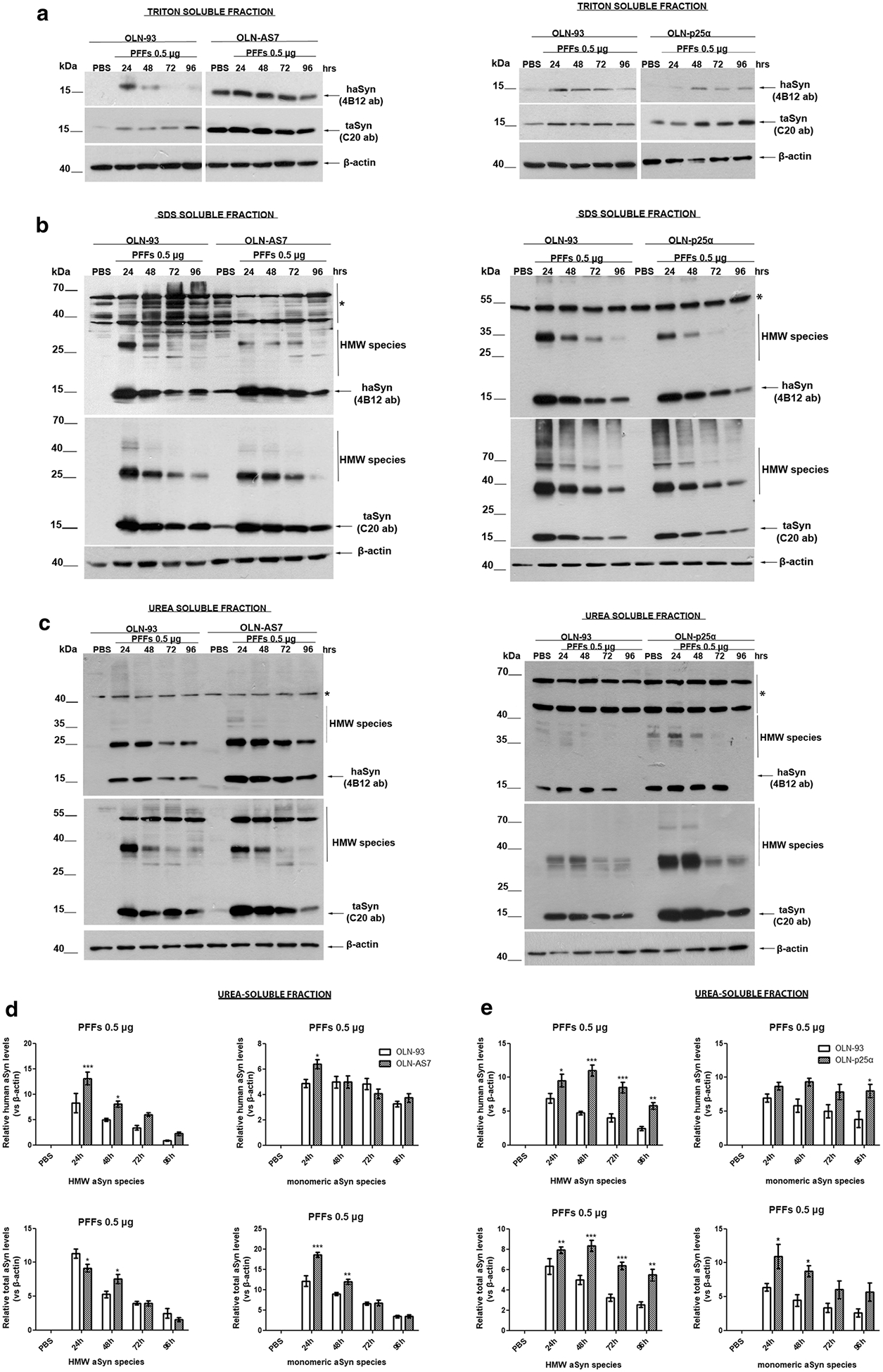
Pre-formed haSyn fibrils are taken up by oligodendroglial cell lines and evoke the formation of Triton-insoluble aSyn species. Representative immunoblots of Triton-, SDS-, and urea-soluble protein fractions derived from OLN-93, OLN-AS7, and OLN-p25α cells treated with 0.5 μg of haSyn PFFs for various times (24–96 h). **a** A small fraction of human and total aSyn (detected with 4B12 and C20 antibodies, respectively) is present in the Triton-soluble fraction of OLN-93 and OLN-AS7 (left panel) or OLN-p25α cell lysates (right panel). **b, c** Monomeric and high molecular weight (HMW) aSyn species (human and total) are mainly detected in the SDS- and urea-soluble fractions, following the addition of haSyn PFFs, thus representing rather insoluble aSyn species in OLN-93 and OLN-AS7 (left panel) or p25α cell lysates (right panel). Equal loading was verified by the detection of β-actin levels. Asterisk represents non-specific bands obtained with the human-specific 4B12 antibody (also detected in the PBS-treated OLN-93 or OLN-p25α cells where no human aSyn was present). **d, e** Quantification of monomeric and HMW species of human (upper panels) and total (lower panels) aSyn levels detected in the urea-soluble fraction of OLN-93 and OLN-AS7 cells (**d**) or OLN-93 and OLN-p25α cells (**e**) treated with 0.5 μg PFFs for 24–96 h. Data are expressed as the mean ± SE of five independent experiments with triplicate samples/condition within each experiment; *p < 0.05; **p < 0.01; ***p < 0.001, by two-way ANOVA with Bonferroni’s correction, comparing between OLN-93 and OLN-AS7 or OLN-p25α cells treated with haSyn PFFs at all time points.

EM used to monitor fibril morphology and to confirm the presence of fibrils (Online Resource 1c). Treatment of OLN-93 cells with 0.2 and 0.5 μg haSyn PFFs for 48 h, led to the dose-dependent accumulation of SDS- and urea-soluble high molecular weight (HMW) aSyn species (Online Resource 1d). We used 0.5 μg haSyn PFFs in subsequent experiments, and we investigated the solubility and aggregation of internalized PFFs in the OLN cell lines over time (24–96 h). The Triton-soluble fraction of all cell lines contained only monomeric aSyn (detected with antibodies against human and human+rodent aSyn) that showed the highest levels at 24 h post-addition, which was followed by a time-dependent decrease to baseline levels (PBS-treated cells), evident mostly in the control OLN-93 cells (Fig. 1a and Online Resource 2a, b). This could be attributed either to the effective clearance of the formed aSyn species or to the dilution of exogenous aSyn due to cell proliferation. Notably, monomeric aSyn levels detected in the Triton-soluble fraction of OLN-AS7 cells remained high over time, possibly due to the stable overexpression of the human protein in these cells, whereas in the OLN-p25α cells both exogenously added human and total aSyn remained at higher levels than in the OLN-93 cells. HMW-aSyn species were detected mostly in the SDS- and urea-soluble fractions (Fig. 1b–c), thus representing rather insoluble aSyn species. Similar to the Triton-soluble fraction, in the SDS-soluble fraction, the peak levels of monomeric and HMW aSyn were observed at 24 h in all cell lines, followed by a gradual decrease over time (48–96 h); however, they remained at higher levels than in the PBS-treated cells (Fig. 1b and Online Resource 2c–f). Interestingly, in the urea-soluble fraction of OLN-p25α cells, aSyn monomeric and oligomeric forms (both human and total) were relatively resistant to degradation, since their levels remained high compared to control OLN-93 cells, in which their levels decreased over time (Fig. 1c–e). This could be indicative of a role for p25α in promoting aSyn aggregate formation within oligodendrocytes or in inhibiting the clearance of aSyn aggregates.

### Addition of haSyn PFFs to OLN cell lines recruits endogenous rat oligodendroglial aSyn into the formation of highly insoluble aSyn conformations

**Fig. 2:**
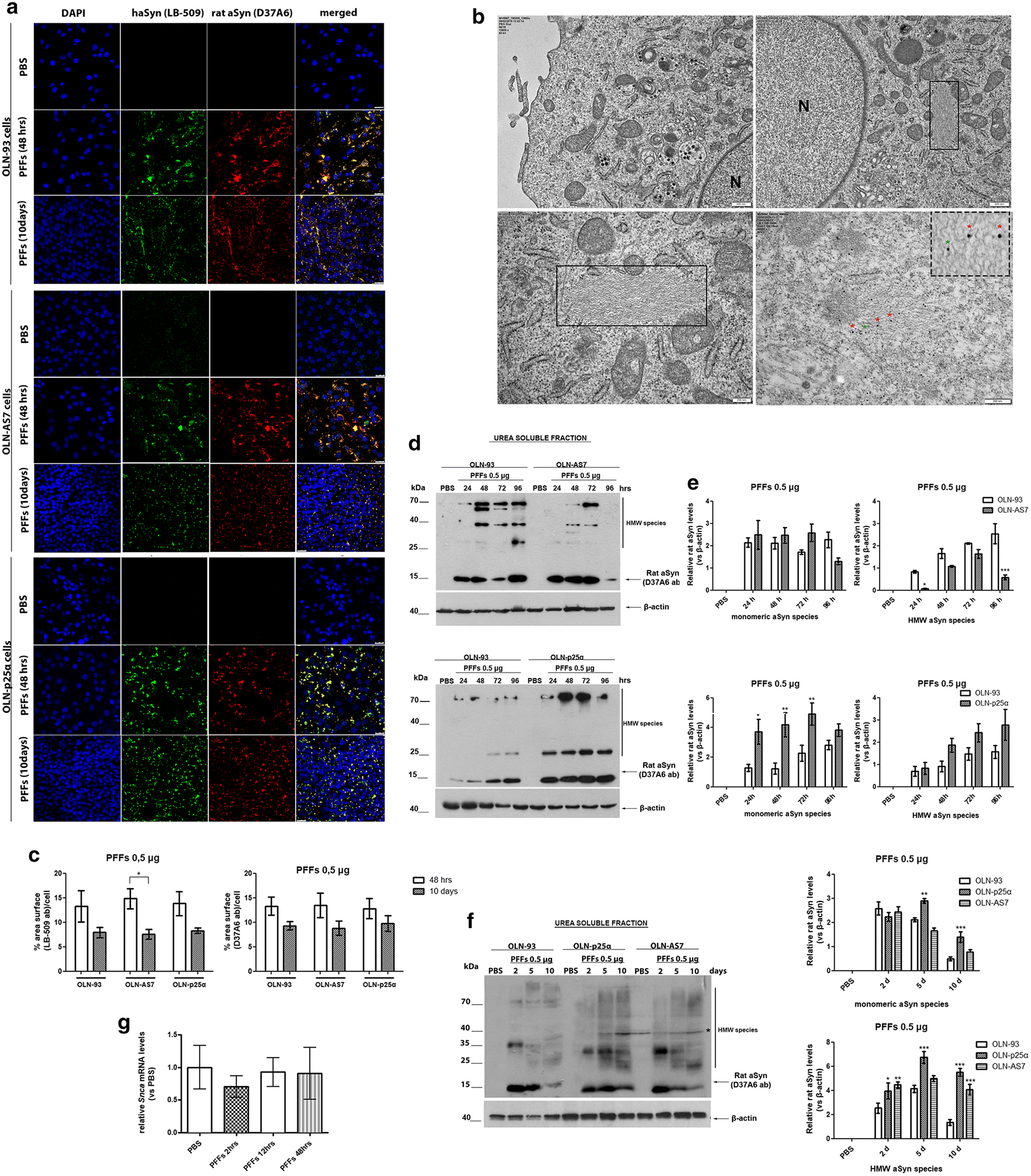
Addition of haSyn PFFs in OLN cell lines leads to the recruitment of endogenous rat oligodendroglial aSyn into highly insoluble aSyn assemblies. **a** Representative immunofluorescence images using antibodies against human aSyn (green, LB509 antibody) and endogenous rat aSyn (red, D37A6 antibody) and DAPI staining in OLN cell lines treated with 0.5 μg of haSyn PFFs for 48 h and 10 days. Scale bar: 25 μm. **b** (upper panels) Ultrastructural transmission EM analysis of PBS-treated (upper left) and haSyn PFFs-treated (upper right) OLN-93 cells showing the presence of filaments (box highlight) in the cell soma near the nucleus of PFF-treated cells (N). (bottom left panel) A higher magnification image of the filaments of PFF-treated cultures is shown. (box highlight, bottom right panel) Double immuno-EM analysis with nanogold particles of different diameters, demonstrating the presence of both human (green asterisk, LB-509, 10 nm) and rodent (red asterisk, D37A6, 15 nm) aSyn in the formed filamentous inclusions in the cell soma of OLN-93 cells. A high-power image of independent PFF-treated cultures is shown in the upper dashed box. Scale bars: 500 nm (upper), 200 nm (bottom) and 100 nm (highlight box). **c** Quantification of aSyn levels (human and endogenous rat) measured as % area surface/cell in OLN cell lines following treatment with 0.5 μg PFFs for 48 h or 10 days. Data are expressed as the mean ± SE of three independent experiments with triplicate samples/condition within each experiment. *p < 0.05 by two-way ANOVA with Bonferroni’s correction. **d** Representative immunoblots demonstrating the presence of endogenous rat aSyn (D37A6 antibody) in the urea-soluble fraction of OLN-93 and OLN-AS7 cells (upper panel) or OLN-p25α cells (bottom panel) following treatment with 0.5 μg haSyn PFFs for 24–96 h. Equal loading was verified by the detection of β-actin levels. **e** Quantification of monomeric (left) and HMW species (right) of endogenous rat aSyn levels detected in the urea-soluble fraction of OLN-93 and OLN-AS7 cells (upper row) or OLN-93 and OLN-p25α cells (bottom row) treated with 0.5 μg PFFs for 24–96 h. Data are expressed as the mean ± SE of five independent experiments with triplicate samples/condition within each experiment. *p < 0.05; **p < 0.01; ***p < 0.001, by two-way ANOVA with Bonferroni’s correction. **f** (left panel) Representative immunoblots of urea-soluble protein fraction derived from OLN-93, OLN-AS7, and OLN-p25α cells treated with 0.5 μg of haSyn PFFs for 2–10 days. Asterisk represents non-specific bands obtained with the rodent-specific D37A6 antibody. (right) Quantification of monomeric (upper row) and HMW species (bottom row) of rat endogenous aSyn detected in the urea-soluble fraction of all OLN cell lines treated with 0.5 μg PFFs for 2–10 days. Data are expressed as the mean ± SE of three independent experiments with triplicate samples/condition within each experiment; *p < 0.05; **p < 0.01; ***p < 0.001, by two-way ANOVA with Bonferroni’s correction, comparing between OLN-93 and OLN-AS7 or OLN-p25α cells treated with haSyn PFFs. **g** Quantitative real-time PCR reveals unaltered *Snca* levels upon treatment of OLN-93 cells with 0.5 μg haSyn PFFs for 2–48 h. Data are expressed as *Snca* mRNA levels relative to the mRNA levels of ß-actin normalized to control-treated cultures (PBS).

In order to investigate the ability of haSyn PFFs to act as a template for endogenous oligodendroglial aSyn to form insoluble protein species, we utilized the rodent-specific D37A6 antibody in western blot and confocal microscopy analyses. This antibody does not give any immunofluorescence signal in PBS-treated OLN cells (Fig. 2a and Online Resource 3); the addition of 0.5 μg haSyn PFFs was accompanied by the detection of an elevated endogenous aSyn signal, which co-localized with the exogenously added human aSyn, that persisted over time (48 h-10 days) and depended, in terms of its intensity, on the exogenously added human material (Online Resource 4). The uptake of haSyn PFFs was further verified by staining the lectins of the plasma membrane using wheat germ agglutinin (Online Resource 1e and 13) and ultrastructural EM analysis, which revealed the presence of filaments in the cell soma of OLN-93 cells treated with haSyn fibrils, which were absent in PBS-treated cells (Fig. 2b, upper panels). Double immuno-EM analysis with nanogold particles of different diameters, showed the presence of human and rat aSyn in the formed intracellular filamentous inclusions (Fig. 2b, bottom panels). Measurement of human or rat endogenous aSyn levels, expressed as % of surface area normalized to the total number of cells/field, revealed no significant differences among the different OLN cells, at 48 h and 10 days post-addition of 0.5 μg haSyn PFFs (Fig. 2c). However, and in agreement with the immunoblot data (Fig. 1c–e), OLN-p25α cells retained significantly more proteinase K-resistant human and rodent aSyn species compared to OLN-93 and OLN-AS7 cells (Online Resource 5).

Similarly, in the urea-soluble fraction of OLN-p25α cells from 24 h to 10 days post-addition of haSyn PFFs, higher levels of monomeric and HMW forms of endogenous rodent aSyn were found compared to OLN-93 and OLN-AS7 cells (Fig. 2d–f). No rat-specific signal was detected in the Triton- or the SDS-soluble fractions at 24h to 10 days post-addition of haSyn PFFs (data not shown). The elevated levels of endogenous aSyn detected following treatment of OLN cells with haSyn PFFs were not attributed to increased transcription of the rat *Snca* gene, encoding endogenous oligodendroglial aSyn, as revealed by quantitative real-time-PCR analysis (Fig. 2g). The specificity of the D37A6 aSyn antibody to recognize only the rodent protein was validated further by confocal microscopy and western immunoblotting, utilizing human SH-SY5Y dopaminergic cells inducibly overexpressing the WT-aSyn upon the removal of doxycycline (dox) [69] and extracts from WT-, KO-, and hA53T-aSyn transgenic mice (Online Resource 6 a, b). Importantly, treatment of OLN-93 cells with the proteasomal inhibitor epoxomicin (epox) or the general lysosomal inhibitor NH_4_Cl alone or in combination (epox+NH_4_Cl) for 48 h, revealed an increased aSyn signal upon lysosomal inhibition, as detected with the D37A6 antibody in the absence of haSyn PFFs, further supporting the presence of miniscule amounts of endogenous aSyn in oligodendrocytes, that are increased upon inhibition of protein degradation (Online Resource 6c, d).

### Overexpression of human aSyn or p25α accelerates the recruitment of the endogenous rat oligodendroglial aSyn and the formation of intracellular oxidized/nitrated aSyn species following the addition of haSyn PFFs

**Fig. 3:**
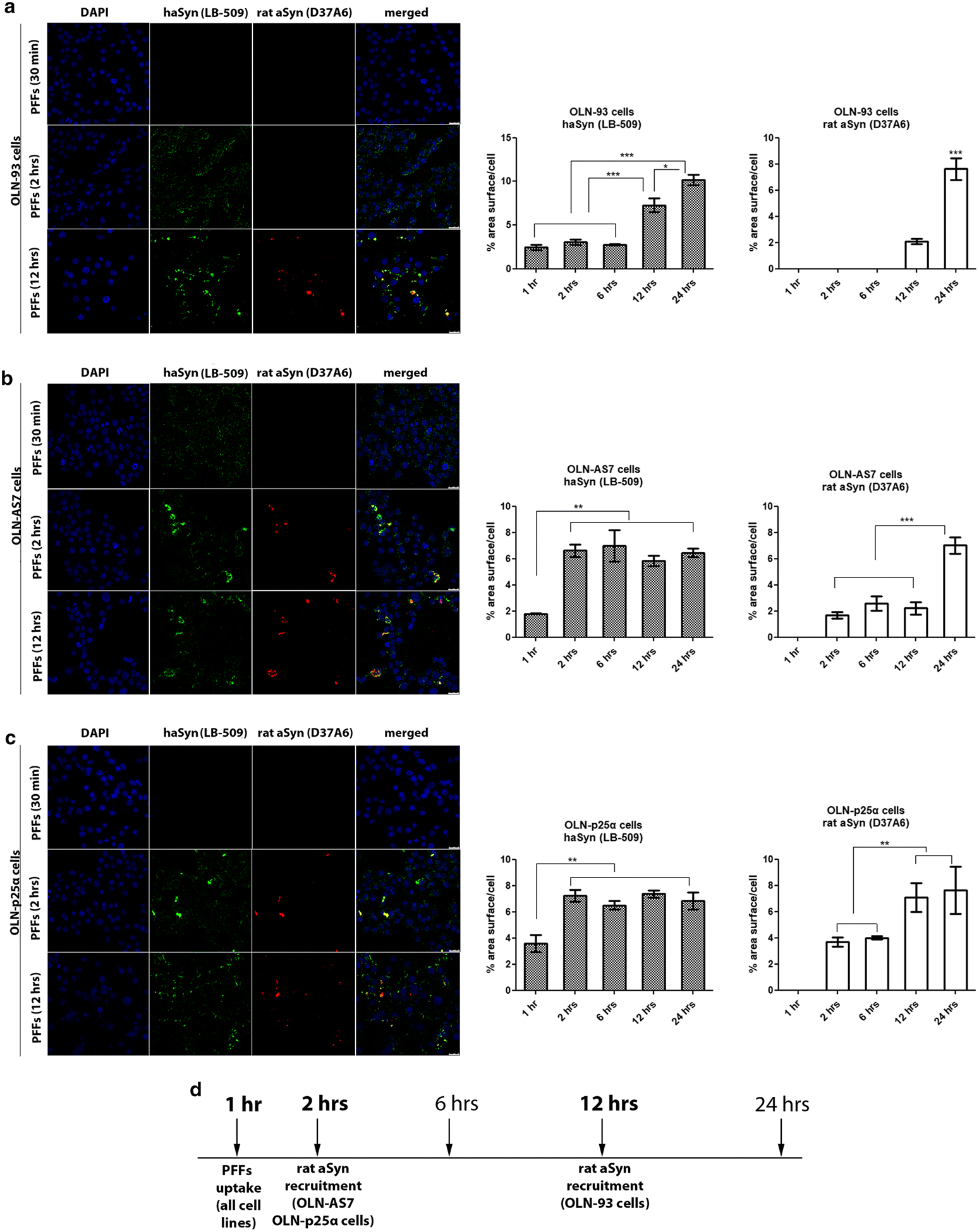
Overexpression of human aSyn or p25α accelerates the recruitment of endogenous rat oligodendroglial aSyn following the addition of haSyn PFFs to OLN cell lines. **a-c** Confocal microscopy with exogenous human-specific and endogenous rat-specific anti-aSyn antibodies reveals the enhanced expression of endogenous rat aSyn at 12 h in control OLN-93 cells and at 2 h in OLN-AS7 and OLN-p25α cells. (left panels) Representative immunofluorescence images with antibodies against human aSyn (green, LB509 antibody) and rodent aSyn (red) and DAPI staining. Scale bar: 25 μm. (right panels) Quantification of aSyn protein levels (human or endogenous rat aSyn) in OLN-93, OLN-AS7, and OLN-p25α cells measured as % area surface/cell following treatment with 0.5 μg haSyn PFFs for various times (2–12 h). Data are expressed as the mean ± SE of three independent experiments with triplicate samples/condition within each experiment; *p < 0.05; **p < 0.01; ***p < 0.001, by one-way ANOVA with Tukey’s post-hoc test. **d** A drawing depicting the key time points when the uptake of exogenous haSyn PFFs and the recruitment of endogenous rat aSyn occur in control cells (OLN-93) and cells overexpressing human aSyn (OLN-AS7) or p25α (OLN-p25α).

To decipher the impact of human aSyn or p25α overexpression on the seeding of aSyn pathology in OLN cells, we performed time-course analysis from 30 min until 24 h post-treatment. Confocal microscopy analysis with antibodies against human and rodent aSyn (LB509 and D37A6 antibodies, respectively) revealed that haSyn PFFs were taken up effectively by all OLN cell lines within 1 h, whereas the recruitment of the endogenous rat aSyn represented a later event (Fig. 3). Interestingly, in control OLN-93 cells, the seeding process was initiated at 12 h post-treatment with the haSyn PFFs (Fig. 3a), whereas in OLN-AS7 and OLN-p25α cells, the endogenous rat aSyn signal was detected earlier, at 2 h post-addition (Fig. 3b–c). Importantly, the recruitment of endogenous rat aSyn evokes a dramatic change in the pattern of haSyn immunostaining and an increase in haSyn immunoreactivity, measured as % area surface/cell, at the onset of seeding (Fig. 3). This observation suggests a possible role for the seeded rat aSyn in the rapid formation of aSyn aggregates positive for both human+rodent aSyn or in the stabilization of haSyn aggregates, rendering them resistant to degradation.

**Fig. 4:**
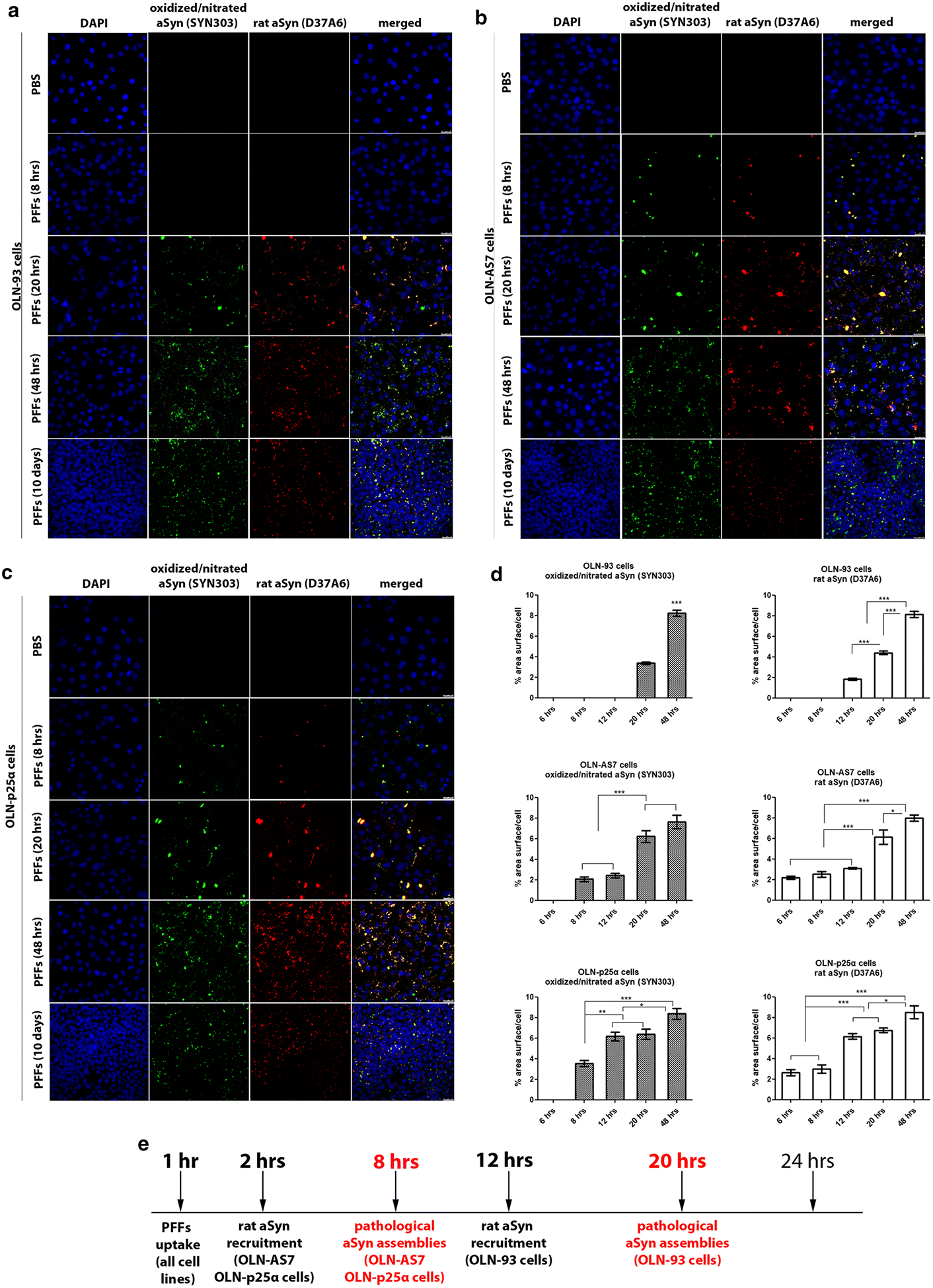
Overexpression of human aSyn or p25α facilitates the formation of pathological (oxidized/nitrated) aSyn assemblies following the addition of haSyn PFFs. **a–c** In OLN-AS7 and OLN-p25α cells, the recruitment of endogenous rat aSyn and the formation of pathological aSyn species are detected at 8 h, whereas they occur in control OLN-93 cells at 20 h post-addition of PFFs. Representative immunofluorescence images of OLN-93, OLN-AS7, and OLN-p25α cells treated with 0.5 μg haSyn PFFs for various times (8–48 h and 10 days) using antibodies against oxidized/nitrated aSyn (green, Syn303 antibody) and endogenous rat aSyn (red) and DAPI staining. Scale bar: 25 μm. **d** Quantification of aSyn protein levels (oxidized/nitrated or endogenous rat aSyn) in OLN-93 (upper row), OLN-AS7 (middle row), and OLN-p25α (bottom row) cells measured as % area surface/cell following treatment with 0.5 μg haSyn PFFs for various times (6–48 h). Data are expressed as the mean ± SE of three independent experiments with triplicate samples/condition within each experiment; *p < 0.05; **p < 0.01; ***p < 0.001, by one-way ANOVA with Tukey’s post-hoc test. **e** A schema highlighting the time points when the increase in endogenous rat aSyn and the detection of pathological (oxidized/nitrated) aSyn species are observed in the different OLN cell lines.

We next investigated whether excess intracellular aSyn protein load (OLN-AS7 cells) or the presence of p25α (OLN-p25α cells) affects the onset of the appearance of aSyn pathology in oligodendrocytes by assessing the formation of oxidized/nitrated aSyn species over time (30 min – 10 days) with confocal microscopy. Indeed, in OLN-AS7 and OLN-p25α cells, the appearance of oxidized/nitrated aSyn (detected with the Syn303 aSyn antibody) was observed within 8 h post-addition of 0.5 μg haSyn PFFs, whereas in the control OLN-93 cells, these pathological species were observed after 20 h (Fig. 4). Notably, co-staining with the rodent-specific aSyn antibody revealed that endogenous aSyn was a major component of the seeded time-resistant pathological aSyn assemblies. Importantly, the Syn303 aSyn antibody did not recognize exogenously added haSyn PFFs *per se*, since no signal was observed at 1 h post-addition, when haSyn PFFs were detected inside the cells (data not shown), further supporting a major role for oligodendroglial aSyn in the seeding of aSyn pathology in OLN cells.

### Transient overexpression of human p25α augments the formation of insoluble aberrant aSyn species, consisting of human and endogenous rat protein

**Fig. 5:**
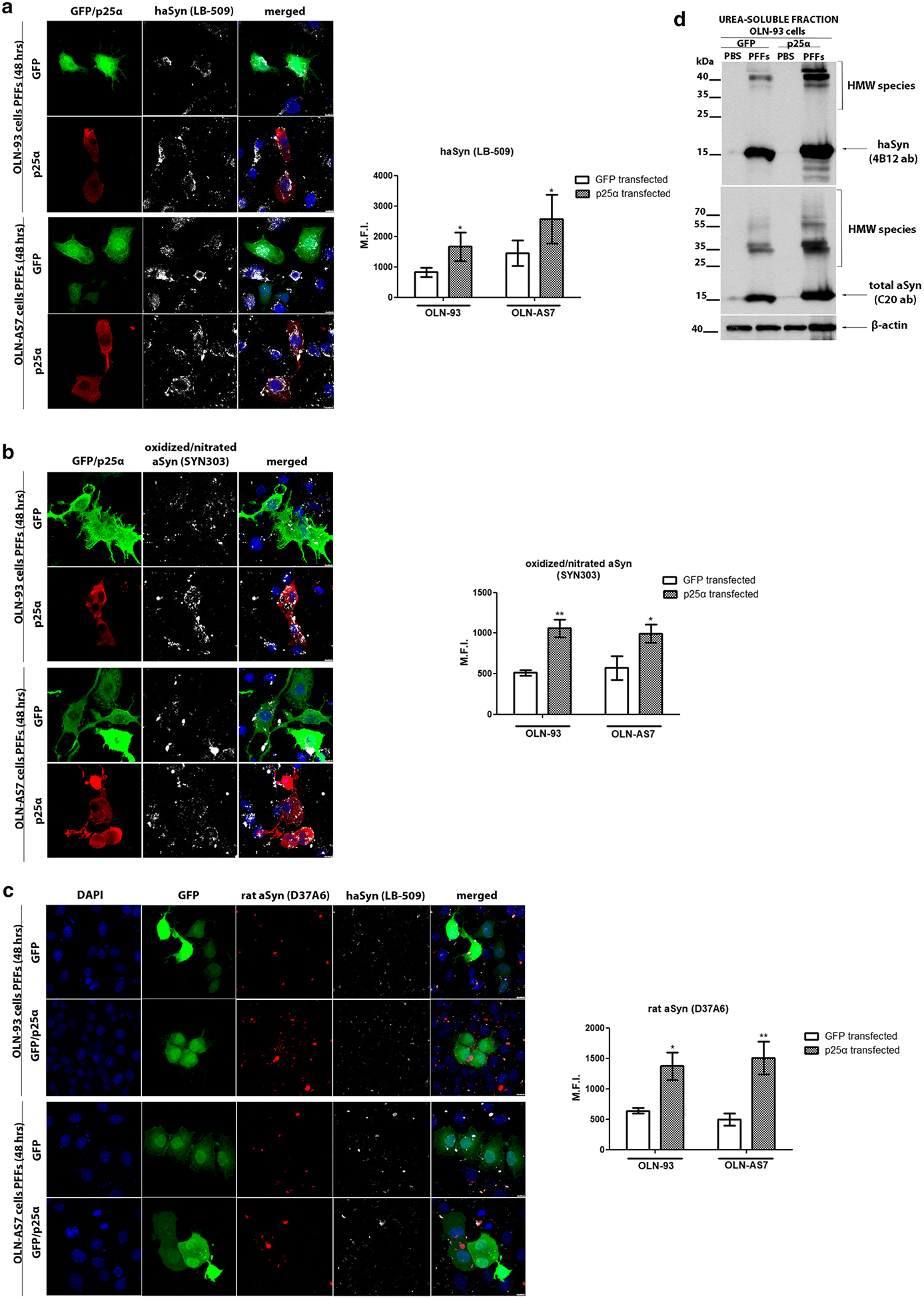
Transient overexpression of human p25α in OLN-93 and OLN-AS7 cells augments the formation of aberrant aSyn species comprised of human and endogenous rat aSyn following the addition of haSyn PFFs. **a–c** (Left panel) Representative immunofluorescence images of OLN-93 and OLN-AS7 cells transfected with GFP or human p25α plasmids in the presence of haSyn PFFs (48 h) using antibodies against: **a** haSyn (gray), **b** oxidized/nitrated aSyn (gray), and **c** rodent aSyn (red). In **a** and **b**, antibodies against GFP (green) and p25α (red) were used to visualize the transfected cells. Scale bar: 10 μm. (Right panel) Quantification of human, oxidized/nitrated, or endogenous rat aSyn protein levels in OLN-93 and OLN-AS7 cells measured as mean fluorescent intensity in selected GFP (green) or p25α (red) transfected cells, using the image analysis tool Imaris (middle panels). Data are expressed as the mean ± SE of at least three independent experiments with triplicate samples/condition within each experiment; *p < 0.05, by Student’s unpaired t-test. **d** p25α overexpression enhances the accumulation of monomeric and HMW aSyn species following the addition of haSyn PFFs. Representative immunoblots of urea-soluble aSyn species of control OLN-93 cells transfected with GFP or p25α plasmids and treated with 0.5 μg haSyn PFFs for 48 h using antibodies against haSyn (4B12 antibody) or total (human+rodent) aSyn (C20 antibody). Equal loading was verified by the detection of β-actin levels.

In order to validate further the role of p25α in the seeding of aSyn pathology, we used another experimental approach. Specifically, OLN-93 and OLN-AS7 cells were transiently transfected (or co-transfected where indicated) with p25α or/and GFP (as a control) plasmids 6 h prior to the addition of 0.5 μg of haSyn PFFs. At 2 days post-transfection, the cells were fixed and processed for confocal microscopy using antibodies against human aSyn (LB509 antibody, Fig. 5a, left panel), oxidized/nitrated aSyn (Syn303 antibody, Fig. 5b, left panel), or rodent aSyn (D37A6 antibody, Fig. 5c, left panel). The transient overexpression of human p25α increased the accumulation of human (Fig. 5a) and endogenous rat (Fig. 5c) aSyn-positive signals and the generation of oxidized/nitrated aSyn species (Fig. 5b), in OLN-93 and OLN-AS7 cells. Additionally, p25α overexpression in OLN-93 cells augmented the formation of monomeric and HMW aSyn species extractable in urea buffer (Fig. 5d). Such data further support a critical role for p25α in the aggregation of pathological aSyn conformations within oligodendrocytes.

### Phosphorylation of aSyn at Ser129 depends on aSyn and p25α protein load and may involve different aSyn aggregate “*strains*” present in oligodendroglial and neuronal synucleinopathies

**Fig. 6:**
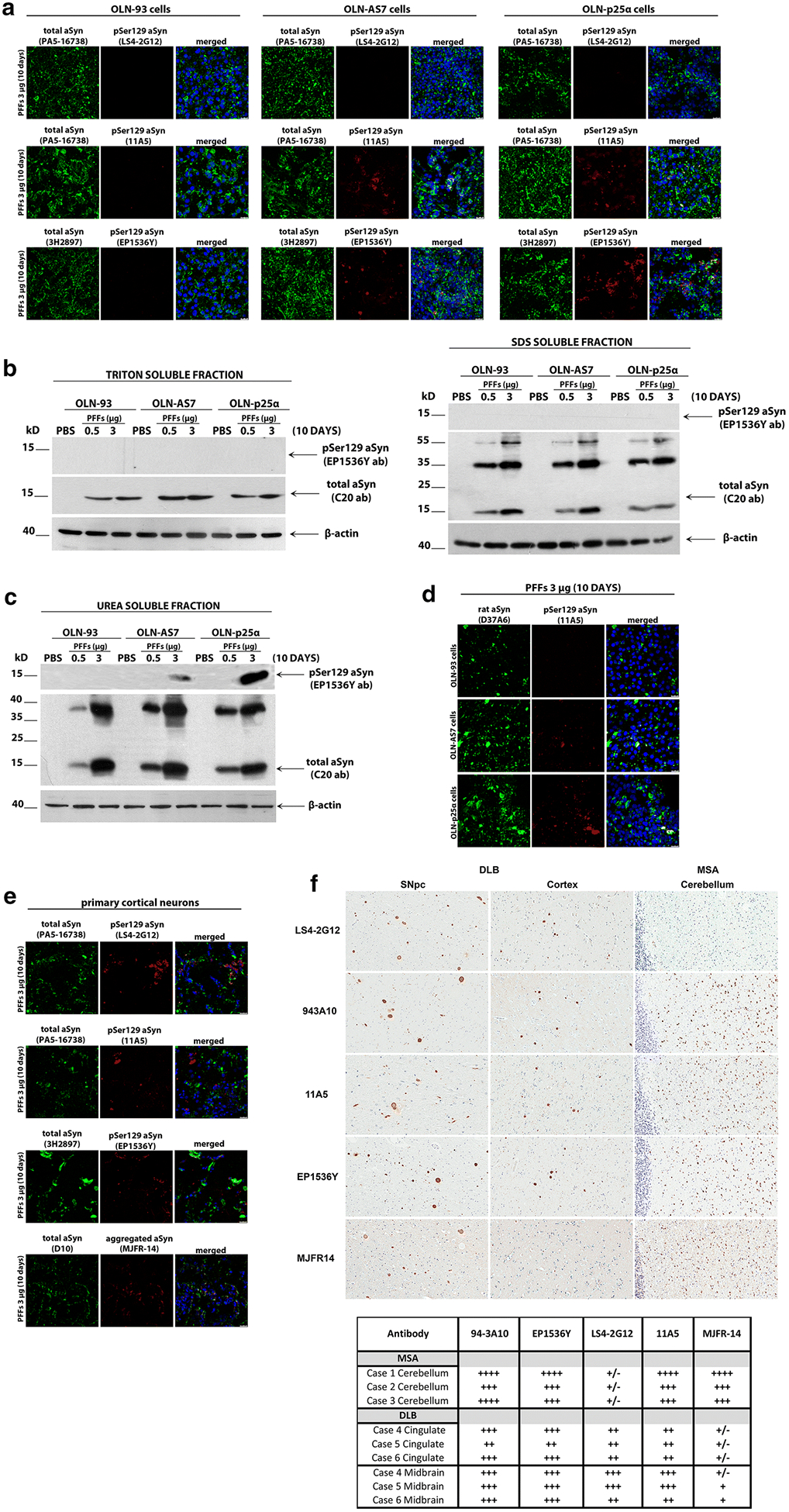
Comparative analysis of pSer129-aSyn phosphorylation in haSyn PFF-treated cultured cells and human post-mortem synucleinopathy brains. **a** OLN-93, OLN-AS7, and OLN-p25α cells were treated with 3 μg haSyn PFFs for 10 days and the phosphorylation of aSyn at Ser129 was examined using various pSer129 aSyn-specific antibodies by confocal microscopy. Representative immunofluorescence images with antibodies against total (human+rodent) aSyn (green, PA5–16738 or 3H2897 antibody) and pSer129 aSyn (red, LS4–2G12, 11A5, or EP1536Y antibody) and DAPI staining. Scale bar: 25 μm. **b, c** Phosphorylated aSyn at Ser129 (EP1536Y antibody) is detected by immunoblotting only in the urea-soluble fractions of OLN-AS7 and OLN-p25α cells following prolonged incubation with 3 μg PFFs. Representative immunoblots of Triton- and SDS-soluble protein fractions (**b**) or urea-soluble protein fractions (**c**) derived from OLN-93, OLN-AS7, and OLN-p25α cells treated with 0.5 μg or 3 μg of haSyn PFFs for 10 days. **d** Endogenous rat aSyn is phosphorylated at Ser129 following prolonged incubation with 3 μg PFFs only in OLN-AS7 and OLN-p25α cells. Representative immunofluorescence images with antibodies against endogenous rat aSyn (green, D37A6 antibody), pSer129 aSyn (red, 11A5 antibody) and DAPI staining. Scale bar: 25 μm. **e** Primary rat cortical neurons were treated with 3 μg haSyn PFFs for 10 days and phosphorylated aSyn, and aggregated aSyn were detected by confocal microscopy. Representative immunofluorescence images with antibodies against total (human+rodent) aSyn (green, PA5–16738, D10, or 3H2897 antibody), pSer129 aSyn (red, LS4–2G12, 11A5, or EP1536Y antibody), aggregated aSyn (red, MJFR-14 antibody) and DAPI staining. Scale bar: 25 μm. **f** (upper panel) Representative DAB-immunostained images using antibodies against phosphorylated (LS4–2G12, 11A5, and EP1536Y antibodies), aggregated aSyn (MJFR-14 antibody) and unmodified aSyn (94–3A10) in human post-mortem brain sections derived from the substantia nigra pars compacta and the cingulate cortex of DLB patients and from the cerebellum of MSA patients. Three confirmed cases of DLB and MSA were utilized for the analysis with similar results, whereby differential reactivity for GCIs vs. LBs was apparent for certain antibodies including LS4–2G12 and MJFR-14. (bottom panel) Immunohistochemical grading of aSyn pathology in different MSA and DLB cases. Glial cytoplasmic inclusions in MSA, cortical LBs in DLB cingulate and brainstem LBs in DLB midbrain were graded using a qualitative assessment of staining intensity and inclusion density by two independent observers. The findings are summarized as (+/−) rare, (+) mild, (++) moderate, (+++) strong, and (++++) severe.

Given that the presence of aSyn phosphorylated at Ser129 (pSer129 aSyn) is an indicator of pathology in synucleinopathies, we assessed pSer129 aSyn levels in all OLN cell lines upon treatment with 0.5 or 3 μg haSyn PFFs for 48 h and 10 days using a panel of specific pSer129 aSyn antibodies. Surprisingly, we detected positive pSer129 aSyn signals only in PFFs-treated OLN cells overexpressing human aSyn or p25α (in the presence of 3 μg haSyn PFFs for 10 days) and not in the control OLN-93 cells, using the pSer129 aSyn-specific antibodies 11A5 (Fig. 6a, middle row) and EP1536Y (Fig. 6a, bottom row). Conversely, no positive signal was detected in any PFF-OLN-treated cell line using the pSer129 aSyn antibody LS4–2G12 (Fig. 6a, upper row), which could be attributed to the low affinity of this antibody. Interestingly, no positive pSer129 aSyn signal was detected in the Triton- or SDS-soluble fraction of all PFF-treated OLN cells (Fig. 6b), whereas pSer129 immunoreactivity was observed only in the urea-soluble fraction of OLN-AS7 cells and to a greater extent in OLN-p25α cells (Fig. 6c). Most importantly, endogenous rat aSyn co-localized with the 11A5 pSer129 aSyn antibody, suggesting that the seeded oligodendroglial aSyn is phosphorylated, upon the addition of haSyn PFFs (Fig. 6d). Strikingly, in PFF-treated primary rat cortical cultures we detected a pSer129 aSyn signal with all antibodies utilized, including the LS4–2G12 antibody that did not give a signal in the OLN seeding model (Fig. 6e), suggesting that this antibody may recognize distinct pSer129 aSyn assemblies generated specifically in neurons and not in oligodendrocytes. Importantly, this hypothesis was further buttressed by data obtained from human post-mortem brain material derived from patients with neuronal synucleinopathy (DLB) and oligodendroglial synucleinopathy (MSA). As shown in Fig. 6f, and in agreement with our results from PFF-treated primary cortical cultures, all pSer129 antibodies recognized pSer129 aSyn in LBs in the substantia nigra pars compacta (left columns) and cortex (middle columns) of DLB brains to a similar extent. In contrast, and congruent with the data obtained from PFF-treated oligodendroglial cells, LS4–2G12 reactivity for MSA cerebellar GCIs was much weaker than the 11A5 and EP1536Y signals. Finally, the conformation-specific aSyn antibody MJFR-14 recognized better the aberrant aSyn species present in GCIs, compared to LBs (Fig. 6f, bottom row), supporting the notion that aSyn generated within oligodendrocytes (MSA) is incorporated into higher order pathological conformations, compared to those generated in neurons (DLB) [8]. In our cellular systems, the MJFR-14 antibody recognized aggregated aSyn in oligodendrocytes (Online Resource 7) and neurons (Fig. 6e, bottom row) to a similar extent, a finding that could be explained by the fact that this antibody also recognizes exogenously added haSyn PFFs.

### Endogenous aSyn is a major component of the seeded pathological aSyn aggregates following the addition of haSyn PFFs

**Fig. 7:**
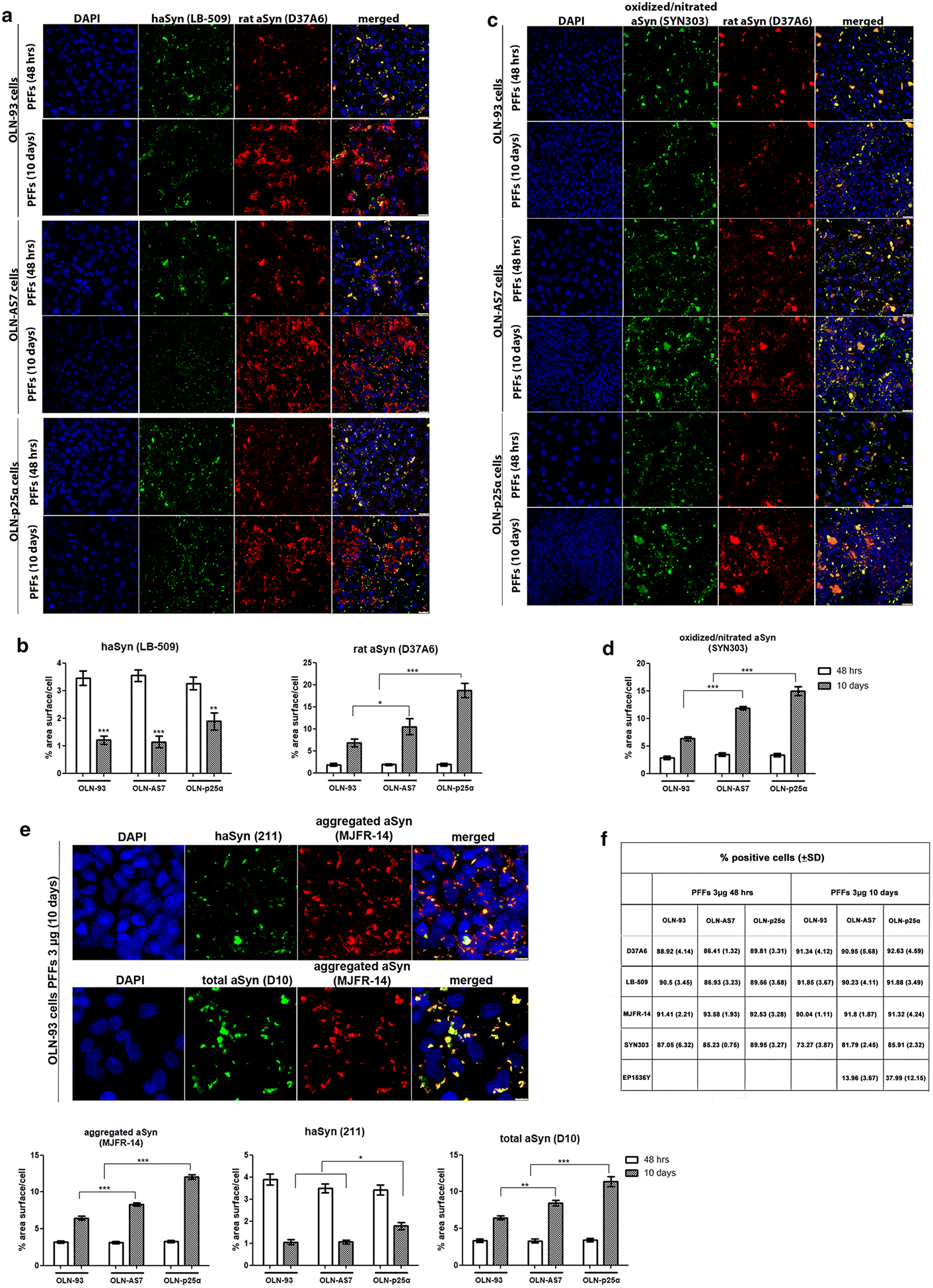
Prolonged incubation of OLN cell lines with high concentrations of haSyn PFFs uncovers the significant contribution of endogenous rat oligodendroglial aSyn to the formation of aberrant aSyn conformations. **a, b** Over time, the equilibrium of aSyn protein load shifts toward seeded endogenous rodent aSyn in all cell lines. OLN-93, OLN-AS7, and OLN-p25α cells were incubated with 3 μg human aSyn PFFs for 48 h or 10 days, and the recruitment of endogenous rat aSyn was examined by confocal microscopy. **a** Representative immunofluorescence images with antibodies against human aSyn (green) and endogenous rat aSyn (red) and DAPI staining. Scale bar: 25 μm. **b** Quantification of human and endogenous rat aSyn protein levels measured as % area surface/cell in OLN cell lines following the addition of 3 μg PFFs for 48 h or 10 days. Data are expressed as the mean ± SE of at least three independent experiments with triplicate samples/condition within each experiment; *p < 0.05; **p < 0.01, ***p < 0.001, by two-way ANOVA with Bonferroni’s correction. **c** The addition of a high concentration of haSyn PFFs (3 μg) leads to the formation of time-resistant pathological (oxidized/nitrated) aSyn species, in which endogenous rat aSyn is the major constituent, as shown using antibodies against oxidized/nitrated aSyn (green) and endogenous rat aSyn (red), and DAPI staining. Scale bar: 25 μm. **d** Quantification of oxidized/nitrated aSyn protein levels measured as % area surface/cell in OLN cell lines following the addition of 3 μg PFFs for 48 h or 10 days. Data are expressed as the mean ± SE of at least three independent experiments with triplicate samples/condition within each experiment; ***p < 0.001, by two-way ANOVA with Bonferroni’s correction. **e** (upper panel) Incubation of OLN cells with 3 μg PFFs for 48 h or 10 days expedites the formation of aggregated conformations of aSyn. Representative immunofluorescence images with antibodies against human aSyn (green, 211 antibody, upper panel), total aSyn (human+rodent, green, D10, bottom panel) and aggregated aSyn (red) and DAPI staining in OLN-93 cells. Scale bar: 7.5 μm. (bottom panels) Quantification of aggregated (left), human (middle), and total (human+rodent) aSyn levels in all OLN cell lines treated with 3 μg haSyn PFFs for 48 h or10 days, expressed as % area surface/cell verifies the aggregated nature of the generated aSyn assemblies and highlights the contribution of recruited endogenous rat aSyn to the formation of such structures. Data are expressed as the mean ± SE of at least three independent experiments with triplicate samples/condition within each experiment; *p < 0.05; **p < 0.01; ***p < 0.001 by two-way ANOVA with Bonferroni’s correction. **f** Quantification of the percentage of positively stained OLN-93, OLN-AS7 or OLN-p25α cells treated with 3 μg PFFs for 48 h or 10 days with antibodies against endogenous rat (D37A60), human (LB-509), aggregated (MJFR-14) or pathological aSyn species (oxidized/nitrated aSyn [Syn303] or pSer129-aSyn [EP1536Y]). All data are expressed as the mean ± SD of at least three independent experiments with triplicate samples/condition within each experiment.

To delineate further the contribution of endogenous oligodendroglial aSyn and p25α in the spread of aSyn pathology, we treated OLN cells with 3 μg PFFs and analyzed aSyn levels at 48 h or 10 days (Fig. 7). With this higher amount of PFFs, over time, the equilibrium of intracellular aSyn protein load in OLN cells shifted toward the seeded endogenous rat protein and notably, this phenomenon was more robust in the presence of human aSyn or p25α (Fig. 7a, b). Similarly, oxidized/nitrated aSyn conformations were elevated in OLN-AS7 and OLN-p25α cells over time (Fig. 7c, d). Finally, using the conformation-specific aSyn antibody MJFR-14, which recognizes aggregated conformations exclusively [27], we detected the induction of MJFR-14-positive structures that colocalized from the earliest time point of 1 h with structures recognized with a pan-aSyn (human+rodent) antibody (D10); the MJFR-14 antibody showed no reactivity against overexpressed aSyn in the OLN-AS7 cell line (Online Resource 7). In order to quantitatively estimate the contribution of aSyn and p25α load to the formation and stability of inclusions, we utilized an indirect immunostaining strategy with antibodies against either total aSyn (D10 antibody) or human aSyn (211 antibody) and aggregated aSyn (MJFR-14 antibody), since both the rodent-specific (D37A6) and MJFR-14 antibodies are generated in rabbits (Fig. 7e). Total aSyn (human and endogenous rat) and aggregate-aSyn fluorescence signals exhibited a nearly complete co-localization (Fig. 7e and Online Resource 7b), whereas staining with the 211 human-specific and MJFR-14 aggregate-specific aSyn antibodies (Fig. 7e and Online Resource 7c) revealed partial co-localization, suggesting indirectly that endogenous rat aSyn is a major component of the formed aggregates. Consistent with this idea, MJFR-14-positive immunostaining increased following the addition of PFFs over a period of 10 days, matching the pattern of endogenous rat aSyn, whereas human aSyn-positive structures remained stable or decreased over time, with OLN-p25α cells again exhibiting higher levels of total and aggregated aSyn (Fig. 7e). Interestingly, the percentage of cells positive for the various aSyn conformations was similar among all OLN cell lines, with the exception of pSer129 aSyn (EP1536Y antibody) (Fig. 7f), suggesting that the observed differences shown in Fig. 7e are possibly attributed to the formation of larger aggregates in aSyn- and p25α-expressing cells, compared to OLN-93 cells. Finally, proof-of-concept experiments utilizing sonicated fibrils yielded similar results regarding the seeding of endogenous aSyn and the formation of pathological assemblies (Online Resource 8).

### Endogenous mouse oligodendroglial aSyn is incorporated into pathological aSyn assemblies in primary oligodendrocytes following the addition of haSyn PFFs

**Fig. 8:**
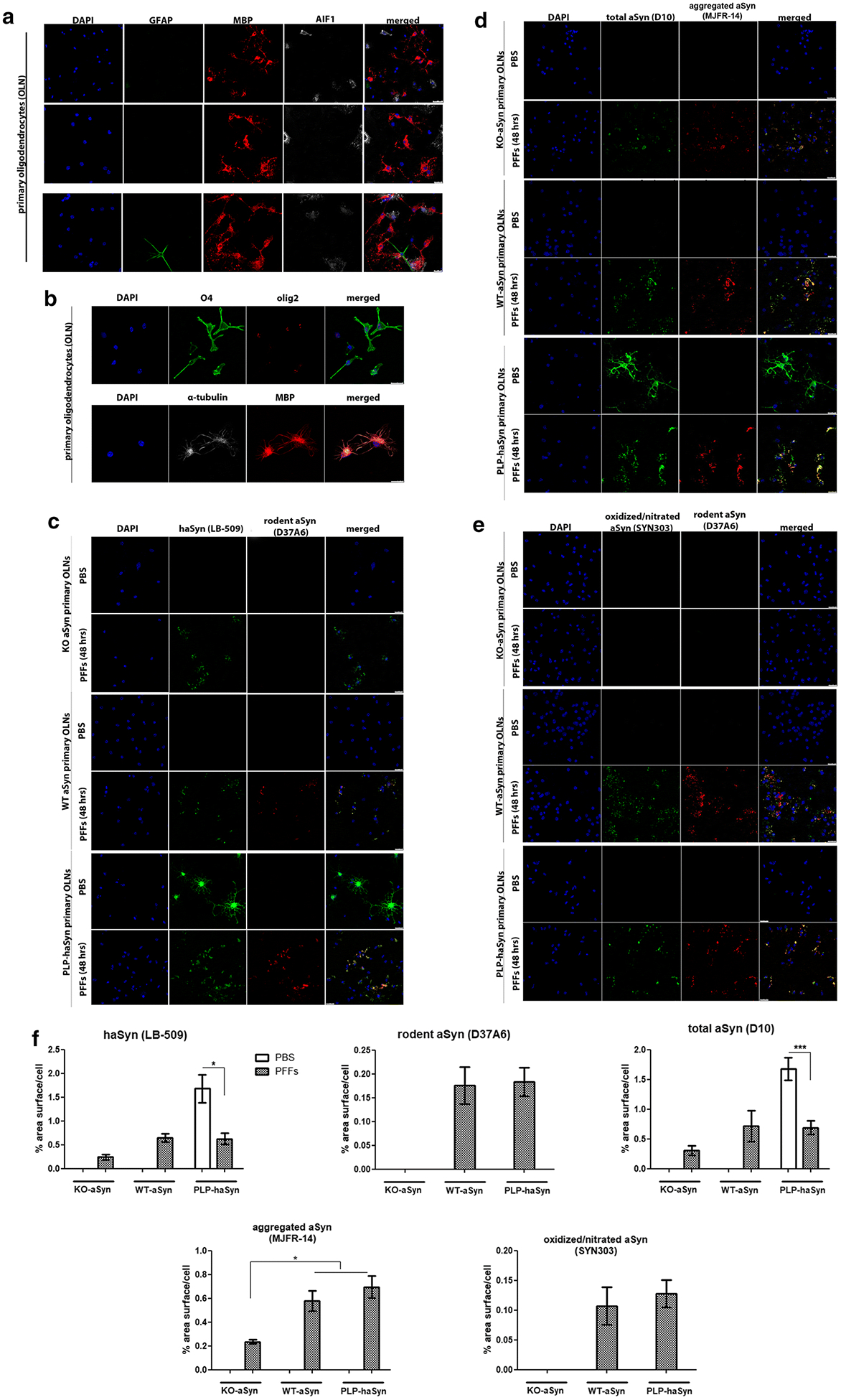
Endogenous mouse oligodendroglial aSyn is incorporated into pathological aSyn assemblies in primary mouse oligodendroglial cultures following the addition of haSyn PFFs. **a, b** Mouse primary oligodendroglial cells derived from WT-aSyn pups were grown in SATO medium for 7 days prior to fixation and immunofluorescent analysis. The enrichment of the cultures in mature myelin-producing oligodendrocytes was confirmed with confocal microscopy using antibodies against glial fibrillary acidic protein as an astrocytic marker (green, GFAP), allograft inflammatory factor 1 (gray, AIF1/IbaI) as a marker for microglia, and myelin basic protein (red, MBP) as a marker for mature oligodendrocytes (**a**), as well as against the oligodendroglial markers Olig2 (red, AB9610 antibody) and O4 (green, MAB1326 antibody) (**b**). α-Tubulin (gray, 62204 antibody) and DAPI staining were used as cytoskeletal and nuclear markers, respectively. Scale bars: 25 μm (**a**, upper panels), 50 μm (**a**, bottom panel) and 10 μm (**b**). **c** Confocal microscopy with human-specific (green) and rodent-specific (red) aSyn antibodies identifies the enhanced expression of endogenous mouse aSyn in WT-aSyn and PLP-haSyn oligodendroglial cultures, upon the addition of 0.5 μg haSyn PFFs for 48 h. No rodent-specific signal is detected in KO-aSyn cells. **d, e** Both exogenously added human aSyn and endogenous mouse aSyn contribute to the formation of aggregated (**d**) and oxidized/nitrated (**e**) conformations of aSyn in WT-aSyn and PLP-haSyn primary oligodendrocytes incubated with haSyn PFFs. Only exogenously added haSyn PFFs (**d**) and not oxidized/nitrated (**e**) conformations are detected in the KO-aSyn cultures. Representative immunofluorescent images using antibodies against total aSyn (green, D10 antibody) and aggregated aSyn (red) are shown in **d** and against oxidized/nitrated aSyn (green) and endogenous mouse aSyn (red) are shown in **e**. Scale bar: 25 μm. **f** Quantification of human, endogenous rodent, and total (human and rodent) aSyn protein levels (upper panel) or aggregated and oxidized/nitrated aSyn protein levels (bottom panel) measured as % area surface/cell in KO-aSyn, WT-aSyn or PLP-haSyn mouse primary oligodendroglial cultures following the addition of 0.5 μg PFFs for 48 h. Data are expressed as the mean ± SE of at least three independent experiments with triplicate samples/condition within each experiment; *p < 0.05; ***p < 0.001, by one-way ANOVA with Tukey’s post-hoc test (to compare between PBS-and PFF-treated cells) or by two-way ANOVA with Bonferroni’s correction (to compare between the different PFF-treated cultures).

To investigate the intracellular processes governing the templating of aSyn pathology in a more physiological cellular setting with properties resembling more closely those of the oligodendrocytes in the central nervous system, we established a protocol to isolate and cultivate mature oligodendrocytes derived from P0-P3 mouse brains. In order to decipher whether the presence of aSyn, which is small in amount, is a prerequisite for the seeding of exogenous added haSyn, we used primary oligodendrocytes prepared from WT- or KO-aSyn mice. In parallel experiments, we utilized primary cultures from PLP-haSyn mice, a well-established MSA mouse model in which haSyn is overexpressed only in oligodendrocytes, under the PLP promoter [20]. To differentiate the oligodendroglial progenitor cells into mature, myelin producing (MBP^+^) oligodendrocytes, the cells were grown in SATO medium. The enrichment of the cultures with oligodendrocytes was verified utilizing markers for astrocytes (GFAP) and microglia (AIF1/IbaI), as well as markers for the oligodendroglial lineage such as Olig2, O4, and MBP (Fig. 8a, b). As observed in the OLN cell lines, the addition of haSyn PFFs (0.5 μg, 48 h) evoked a dramatic increase in the levels of endogenous mouse oligodendroglial aSyn only in the WT-aSyn and PLP-haSyn primary cultures (Fig. 8c). No fluorescence signal for the rodent-specific D37A6 aSyn antibody was detected in the KO-aSyn cultures or at baseline in the WT- aSyn and PLP-haSyn cultures in the absence of haSyn PFF treatment (Fig. 8c, f). The magnitude of the increase was dependent on the endogenous aSyn load, since the rodent-specific aSyn signal was more intense and more compact in the PLP-haSyn oligodendrocytes than in WT-aSyn oligodendrocytes (Fig. 8c). The formed aSyn species were aggregated, as verified by the strong immunoreactivity of the conformation-specific MJFR-14 antibody, which co-localized completely with the D10 antibody, recognizing the human+rodent protein (Fig. 8d). Interestingly, the expression pattern of aSyn in the haSyn-PLP cultures changed dramatically from diffuse (in PBS-treated cultures) to strongly punctated (in PFF-treated cultures), as detected by the LB509 and D10 antibodies (Fig. 8c, d). Finally, the rodent-specific-positive puncta colocalized to a great extent with the oxidized/nitrated aSyn antibody in the WT-aSyn and haSyn-PLP cultures, but not in the KO-aSyn cultures (Fig. 8e, f), further indicating that the endogenous protein contributes to the formation of such pathological conformations.

### Endogenous oligodendroglial aSyn protein load is closely linked to the re-distribution of microtubule-associated proteins upon the addition of haSyn PFFs

**Fig. 9:**
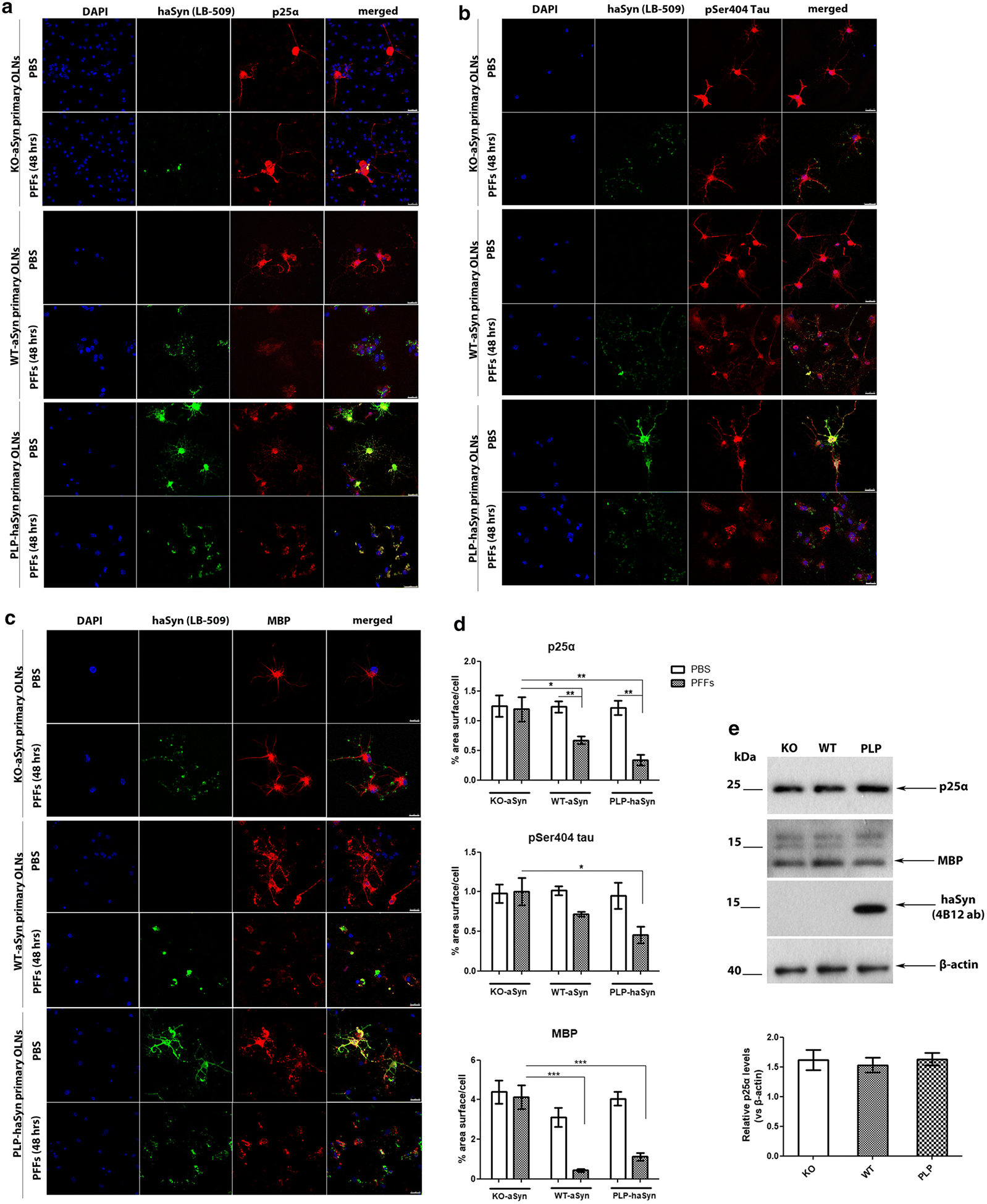
The re-distribution of the microtubule-associated proteins p25α, tau, and MBP in primary oligodendroglial cells depends on endogenous aSyn protein load following the addition of haSyn PFFs. **a** p25α redistributes from the oligodendroglial processes to the cell body only in WT-aSyn and in PLP-aSyn cultures, and is trapped in aSyn-positive aggregates only in PLP-haSyn oligodendroglial cells (bottom rows), following treatment with haSyn PFFs for 48 h. No relocalization of p25α is observed in KO aSyn primary oligodendroglial cultures (upper rows). Representative immunofluorescence images with antibodies against human aSyn (green, LB509), endogenous mouse p25α (red, p25α antibody) and DAPI staining. Scale bar: 25 μm. **b, c** The cellular pattern and distribution of phosphorylated tau at residue Ser404 and MBP changes dramatically following the addition of haSyn PFFs in a manner dependent upon the endogenous aSyn protein load. No differences are observed in KO-aSyn cultures treated with haSyn PFFs, thus highlighting the contribution of the endogenous oligodendroglial aSyn to the dysregulation of myelin. Immunofluorescence images with antibodies against human aSyn (green) and pSer404 tau (red, AP01708PU-N antibody) are shown in **b** and against human aSyn (green) and MBP (red) are shown in **c**. DAPI is used as a nuclear marker. Scale bar: 25 μm. **d** Quantification of p25α (upper row), pSer404 tau (middle row), or MBP protein levels (bottom row) in KO-aSyn, WT-aSyn, and PLP-haSyn primary oligodendrocyte cultures treated with 0.5 μg haSyn PFFs for 48 h measured as % area surface/cell. Data are expressed as the mean ± SE of three independent experiments with three samples/condition within each experiment; *p < 0.05; **p < 0.01; ***p < 0.001, by one-way ANOVA with Tukey’s post-hoc test (to compare between PBS-and PFF-treated cells) or by two-way ANOVA with Bonferroni’s correction (to compare between the different PFF-treated cultures). **e** No differences are detected in p25α protein levels expressed in KO-aSyn, WT-aSyn, or PLP-haSyn primary oligodendrocytes. (upper panel) Representative immunoblots of p25α, MBP and haSyn (4B12 antibody) protein levels are shown. Equal loading was verified by the detection of β-actin levels. (bottom panel) Quantification of p25α protein levels expressed in all primary oligodendroglial cultures. Data are expressed as the mean ± SE of three independent experiments with triplicate samples/condition within each experiment.

Given the close link between aSyn levels and the redistribution p25α in disease pathogenesis, we assessed the expression pattern of p25α in PFF-treated primary mouse oligodendrocytes. As shown in Fig. 9a and 9d, there was no apparent change in the pattern of p25α expression in PFF-treated KO-aSyn cultures (upper rows), in contrast to WT-aSyn cultures, in which p25α was redistributed from the oligodendroglial processes to the cell body, although no obvious co-localization with haSyn-positive aggregates was observed (middle rows). Remarkably, p25α in the PFF-treated PLP-haSyn oligodendrocytes, beyond its cell body redistribution, accumulated clearly in haSyn-positive cytoplasmic aggregates (bottom rows). This change was related to haSyn PFFs, given that p25α protein levels were similar between the untreated oligodendroglial cultures (Fig. 9a, d, e).

Since p25α is a tubulin polymerization-promoting protein that is involved in the organization of the microtubule network [65], its re-localization in the presence of haSyn PFFs might also be linked to the altered localization of other microtubule-associated proteins, such as tau, which has also been found to be present in GCIs [64]. To investigate whether the distribution pattern of tau was affected upon the addition of haSyn PFFs, we studied tau phosphorylated at residue Ser404 (pSer404-tau), which is one of the first sites to be hyperphosphorylated leading to the detachment of tau from microtubules, a phenomenon that is closely related to disease pathogenesis in tauopathies [1, 16, 36, 63]. Our data showed that pSer404-tau was redistributed from the processes to the cell soma upon the addition of haSyn PFFs in a manner dependent on the endogenous aSyn load, since this effect was more evident and robust in PLP-haSyn cultures, whereas the pSer404-tau immunostaining pattern remained unchanged only in the case of KO-aSyn primary oligodendrocytes (Fig. 9b, upper panel and 9d). However, pSer404-tau largely failed to be incorporated into haSyn aggregates, even in PLP-haSyn cultures, revealing a different behavior compared to p25α; this may have occured because this specific phosphorylated form, in contrast to p25α, is unable to bind to microtubules.

Finally, we assessed the impact of exogenously added haSyn PFFs on myelination, which is impaired in MSA brains [10, 12, 61]. The addition of haSyn PFFs to primary oligodendrocyte precursor cells reportedly leads to the disruption of MBP expression [21]. However, the role of endogenous aSyn in the levels and distribution of MBP remains unknown. In order to investigate whether alterations in the levels of endogenous oligodendroglial aSyn may be linked to alterations in myelin integrity, we assessed haSyn and MBP by confocal microscopy. Our data showed that the presence of endogenous mouse aSyn (in WT-aSyn cultures) or overexpressed human aSyn (in PLP-haSyn cultures) led to a dramatic alteration of MBP immunoreactivity following the addition of haSyn PFFs; in particular, MBP staining was drastically decreased and redistributed from the processes to the cell soma, largely colocalizing with aggregated haSyn (Fig. 9c, d). On the contrary, KO-aSyn mouse oligodendrocytes did not display any significant changes in MBP levels or distribution (Fig. 9c, upper panel), thus indicating a crucial role for seeded oligodendroglial aSyn aggregation in the alterations of MBP.

### Delivery of haSyn PFFs into the mouse striatum is accompanied by the recruitment of endogenous oligodendroglial protein in MBP^+^ pathological aggregates and the impairment of myelin integrity

**Fig. 10:**
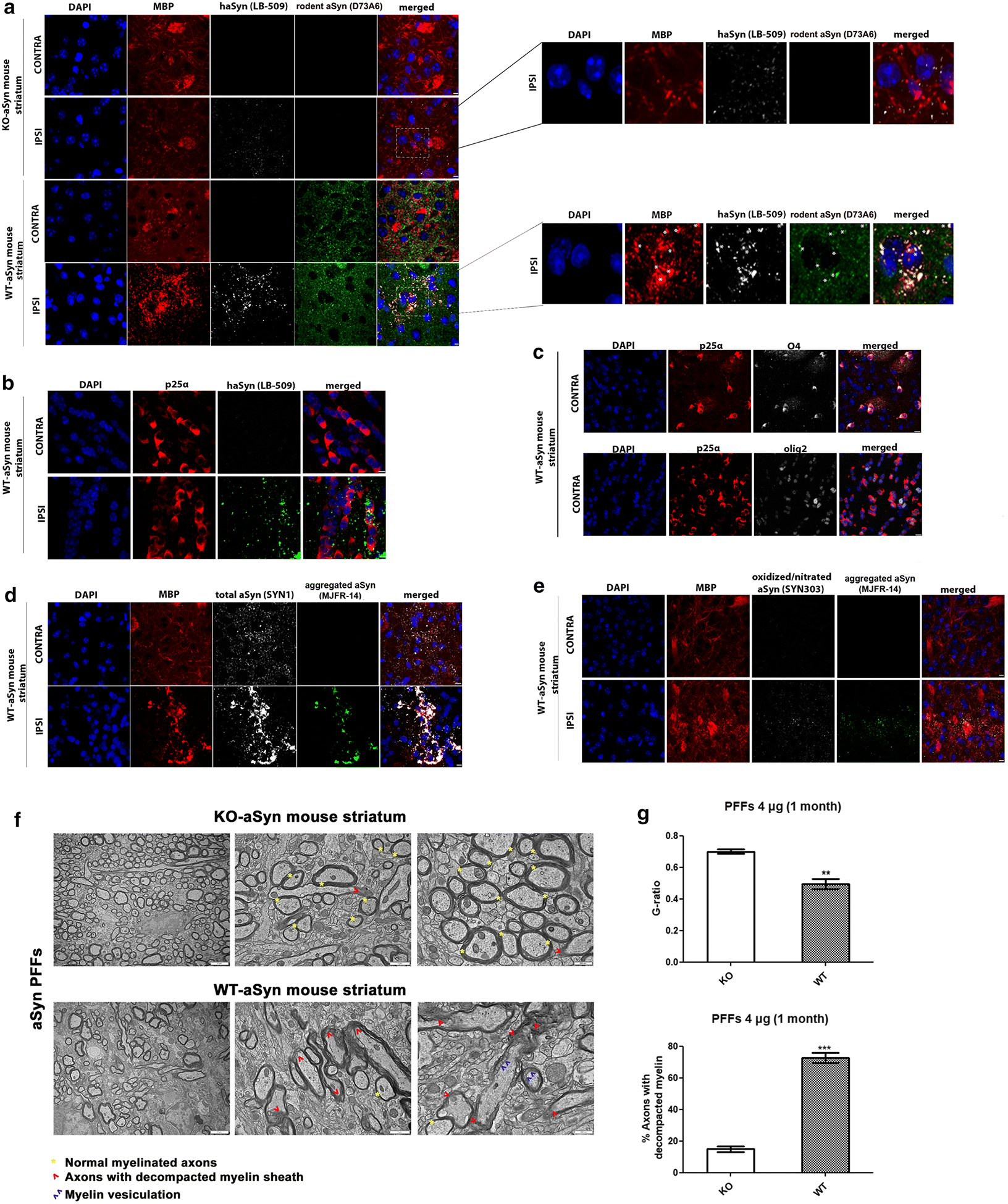
Unilateral delivery of haSyn PFFs (4 μg) into the WT-aSyn mouse striatum results in the recruitment of endogenous mouse oligodendroglial aSyn and the formation of pathological aSyn assemblies. **a** Representative immunofluorescence images showing the co-localization (indicated by the asterisks) of exogenously added human aSyn PFFs (gray) with endogenous mouse aSyn (green) in MBP^+^ cells (red) in the ipsilateral (IPSI) striatum, but not in the contralateral (CONTRA) striatum, at 1 month post-injection (lower panel). Importantly, no endogenous mouse-specific aSyn signal was detected in the ipsilateral striatum of KO-aSyn mice (upper panel). Scale bar: 10 μm. High-power merged images are shown on the right. **b** Human aSyn PFFs are taken up by striatal oligodendrocytes *in vivo*, as shown by confocal microscopy using antibodies against human aSyn (green, LB-509 antibody) and the oligodendroglial marker p25α (red, p25α antibody). Scale bar: 10 μm. **c** The specificity of our home-made p25α antibody to oligodendrocytes was further verified using the O4 (gray, upper panel) and Olig2 (gray, bottom panel) antibodies. Scale bar: 10 μm. **d** MBP redistributes in the PFF-injected striatum and co-localizes with aggregated aSyn conformations. Representative immunofluorescence images for MBP (red), human aSyn (gray, SYN1 antibody), aggregated aSyn (green) and DAPI staining. Scale bar: 10 μm. **e** Pathological aSyn assemblies are detected in the ipsilateral striatum of WT-aSyn mice injected with haSyn PFFs at 1 month post-injection, as verified using antibodies against oxidized/nitrated aSyn (gray) and aggregated aSyn (green). Scale bar: 10 μm. **f** Ultrastructural transmission EM analysis of haSyn PFF-injected KO-aSyn (upper panel) or WT-aSyn (bottom panel) mouse striatum at 1 month post-injection, revealing myelin sheath decompaction only in WT-aSyn neuronal axons (red arrowheads) vs. normal myelinated axons (yellow asterisks). Purple arrowheads point to areas with vesiculated myelin sheaths. Scale bars: 2 μm (left panels), 1 μm (middle panels), and 500 nm (right panels). **g** Average myelin g-ratio values (upper row) and quantification of axons with decompacted myelin (bottom row) derived from haSyn PFF-injected KO-aSyn or WT-aSyn mouse striatum. Data are expressed as the mean ± SE of n=4 mice per group. **p < 0.01; ***p < 0.001 by Student’s unpaired t-test.

In order to evaluate the seeding capacity of haSyn within oligodendrocytes *in vivo*, we performed unilateral stereotactic injections of haSyn PFFs (4 μg/brain) (or PBS as a control) into the mouse striatum, to imitate the situation occurring in MSA brains. As shown in Fig. 10a (bottom panels), the delivery of haSyn PFFs into the mouse striatum seeded the recruitment of endogenous mouse aSyn to form MBP-positive inclusions, which were positive for human and rodent aSyn, detected at 1 month post-injection (Fig. 10a, IPSI, indicated by the asterisks). The uptake of haSyn PFFs by oligodendrocytes was verified further by co-staining with the p25α antibody (Fig. 10b and Online Resource 15), whose specificity for oligodendrocytes in the mouse striatum was confirmed by its complete co-localization with O4 and Olig2 antibodies (Fig. 10c).

Consistent with the results in the primary oligodendroglial cultures, the MBP-positive signal changed dramatically upon the addition of haSyn PFFs, and MBP^+^ puncta colocalized with the human (LB-509), rodent (D37A6), total (SYN1) and aggregated aSyn antibodies (Fig. 10a, d and Online Resource 14). Importantly, such alterations were not observed when a similar amount of haSyn PFFs was injected into the striatum of KO-aSyn mice (Fig. 10a, upper panels). Staining with the Syn303 aSyn antibody, which recognizes oxidized/nitrated species, revealed the formation of pathological aggregated aSyn assemblies in MBP^+^ oligodendrocytes of the ipsilateral striatum within 1 month of injection (Fig. 10e). No Syn303 aSyn-positive signal was detected in haSyn PFF-injected KO-aSyn striatum (data not shown), and none of the aforementioned pathological alterations were observed in the ipsilateral striatum of WT-aSyn mice injected with PBS (Online Resource 9). Importantly, ultrastructural EM analysis revealed that haSyn PFFs evoked a clear rearrangement of the myelin sheath in the striatum of WT-aSyn mice, compared to PFF-treated KO-aSyn mice, as depicted by the increased number of axons with a decompacted myelin sheath and axons with myelin vesiculation (Fig. 10f, g). Such data support further the aforementioned results in PFF-treated primary oligodendrocytes (Fig. 9c, d) and in the PFF-treated ipsilateral WT-aSyn striatum (Fig. 10a, d).

## Discussion

Two possible scenarios have been proposed to explain the origin of aSyn in oligodendrocytes and the mechanisms underlying aSyn accumulation in GCIs present in MSA brains: either oligodendrocytes pathologically overexpress aSyn in the context of MSA [3] or they take up neuronally derived protein from their environment [13, 21, 23, 24, 48, 49, 55, 58]. Our results combine these two scenarios by pinpointing the accumulation of endogenous oligodendroglial aSyn as a critical factor for the generation of pathological GCI-like aSyn structures within oligodendrocytes *in vitro*, triggered by exogenously added human fibrillar aSyn seeds. We also report that the oligodendroglial phosphoprotein p25α plays a pivotal role in the development and progression of aSyn aggregation, by modulating the rate and extent of aSyn seeding and, to some extent, by being incorporated into aSyn-rich pathological assemblies. Notably, the incorporation of endogenous oligodendroglial aSyn in such aberrant inclusions and the impairment of myelin integrity were demonstrated in the PFF-injected mouse brain, thus further highlighting the contribution of the endogenous protein in aSyn pathology in MSA-like models.

MSA, Parkinson’s disease (PD), and DLB are collectively termed synucleinopathies [39, 41, 62]. In contrast to PD and DLB, in which aSyn accumulates in neurons that physiologically express the protein, in MSA, the pathological accumulation of abnormally folded aSyn is found mainly in oligodendrocytes [66]. Neuronal cytoplasmic and nuclear aSyn inclusions and dystrophic neurites can also be present in MSA brains; however, these lesions appear at a lower frequency than GCIs [40]. Initial studies suggested that oligodendrocytes do not express aSyn [35, 38, 60], whereas subsequent reports proposed that it may be expressed in oligodendrocytes, albeit at lower levels than in neurons [3, 9]. However, the prevailing hypothesis based mostly on *in vitro* data is that neurons secrete aSyn, which is subsequently taken up by surrounding oligodendrocytes [13, 21, 23, 24, 48, 49, 55, 58].

Herein, we assessed the capability of templating the misfolding of endogenous protein, to examine the hypothesis that the prion-like transformation of oligodendroglial aSyn can occur, as has been demonstrated for its neuronal counterpart [2, 29, 30, 59, 72]. Our results show that exogenously added haSyn PFFs are taken up by immortalized oligodendroglial cells and primary oligodendrocytes and can induce the formation of highly insoluble and pathological (aggregated, oxidized/nitrated, and under certain circumstances Ser129 phosphorylated) aSyn species in which the endogenous protein, however minute in amount at baseline, is a central component. The mechanism responsible for the tremendous increase in the expression of endogenous rodent aSyn following PFF addition remains unknown; we have found that the levels of rodent *Snca* mRNA are unchanged in this setting, consistent with the results reported by others in oligodendroglial progenitor cells [21], suggesting that the stabilization of oligodendrocyte aSyn may be involved. One possible scenario is that the catabolism of aSyn, most probably through the autophagy pathway as suggested by the accumulation of oligodendroglial aSyn upon lysosomal inhibition, is abrogated by the formation of aberrant aSyn species following the addition of haSyn PFFs.

An upsurge in the expression of endogenous aSyn in haSyn PFF-treated oligodendroglial progenitor cells in culture and the altered expression of proteins associated with neuromodulation and myelination were recently reported [21]. However, the authors of that study failed to find evidence that a similar process occurs in mature, myelin-forming oligodendrocytes. Herein, we show clearly, in contrast to these data, that the addition of haSyn PFFs to mature differentiated mouse primary oligodendrocytes induces the seeding of endogenous rodent aSyn, and the dysregulation in myelin, as manifested by the decreased levels and perisomal redistribution of MBP, as well as the colocalization of MBP within aSyn-positive aggregates. This colocalization upon of haSyn PFF administration is similar to what is proposed to occur during the formation of GCIs in human MSA [74]. Such a disruption of the myelin sheath is responsible for demyelination, which contributes to disease pathology and clinical manifestations [12, 31, 61, 77]. Remarkably, this disruption of myelin was completely prevented in KO-aSyn cultures and in the PFF-injected KO-aSyn striatum, indicating that the presence of endogenous oligodendroglial rat aSyn is necessary for the manifestation of this pathological hallmark of MSA. Furthermore, p25α and phospho-tau staining in PFF-treated cultures demonstrated a disruption of the microtubule network that was dependent on the levels of endogenous aSyn. In agreement with our findings, Grigoletto et al. showed an age- and disease-dependent loss of the MBP signal in striatal striosomes accompanied by reduced p25α levels in oligodendrocytes in neuronal synucleinopathy mouse models [15]. They concluded that neuronal aSyn is involved in the regulation and/or maintenance of myelin phospholipids, since aSyn also inhibited the maturation of oligodendrocytes and the formation of membranous sheets *in vitro* [15].

Toward the same direction, our *in vitro* data suggest a role for p25α in the prion-like seeding process and aggregation of endogenous oligodendroglial aSyn, illustrating that both the stable and transient overexpression of p25α accelerated the seeding of endogenous aSyn and augmented the formation of pathological aSyn aggregates. p25α reportedly plays a critical role in myelin maturation and relocates early from the myelin sheath to the cytoplasm in MSA [61]. This relocation may impair myelin synthesis and seed aSyn aggregation in oligodendrocytes. A recent study also showed that p25α not only relocates from peripheral processes but also from the nucleus to the perinuclear cytoplasm in MSA patients [37]. The mechanism by which p25α enhances aSyn seeding and aggregation is unclear, and our data suggest it does not seem to involve necessarily the direct incorporation of p25α into aSyn aggregates, as this occurs only in the case of PLP-haSyn primary oligodendrocytes. Moreover, the absence of p25α redistribution in PFF-treated KO-aSyn cultures, suggests that the presence of endogenous oligodendroglial aSyn is a prerequisite for such a phenomenon to occur. Hinting at a possible mechanism, preliminary experiments in our laboratory using OLN-AS7 cells showed that transient p25α overexpression triggered the phosphorylation aSyn at Ser129 and increased the total levels of aSyn (unpublished observations). Similarly, the co-expression of haSyn and p25α results in the phosphorylation of aggregated, but not monomeric aSyn, in OLN-AS7 cells [26]. Consistent with this idea, in the current work, p25α appeared to stabilize insoluble aSyn conformations. These findings may be applicable more broadly to alpha-synucleinopathies, since p25α is reportedly ectopically expressed in dopaminergic neurons in PD and to be a component of LBs purified from human post-mortem PD brains [28].

Research over the last few years using recombinant aSyn PFFs and samples from patients with synucleinopathy has provided support to the hypothesis that aSyn may misfold and propagate in a prion-like fashion in MSA patients [17]. The injection of brain homogenates containing aSyn derived from MSA patients into mice triggers the formation of phosphorylated aSyn aggregates and the onset of a neurodegenerative cascade comparable to human MSA pathology [4, 47]. Moreover, recent reports have shown that the aSyn pathological conformations found in MSA brains are remarkably stable and resistant to inactivation [75] and that MSA is caused by a unique strain of aSyn assemblies [34, 44, 47]. Supportive of the latter hypothesis are findings showing that pathological aSyn in GCIs and LBs (detected by the Syn303 and Syn7015 fibril-specific antibodies) is conformationally and biologically distinct and that oligodendrocytes, but not neurons, transform misfolded aSyn into a GCI-like strain, highlighting the fact that distinct aSyn strains are generated in different intracellular milieus [45]. Our comparative analyses of the phosphorylation status of aSyn utilizing different pSer129-specific antibodies in oligodendroglial cells, primary cortical neurons, and human post-mortem material from DLB and MSA brains, further favor the idea that synucleinopathies are caused by different aSyn strains leading to the detection of distinct aSyn pathological conformations in each disease, which depend on the intracellular context [34, 44, 45].

Along these lines, it is important that we assessed aSyn pathology exclusively within the context of oligodendroglial cell lines, primary mature oligodendrocytes, and even *in vivo* within defined oligodendroglial cells *in situ*. Of course the present study has limitations that merit consideration, given that we have utilized uniquely synthetic haSyn PFFs to induce aSyn aggregation, an approach used widely in the field [46, 71, 72]. Another caveat is that we employed human aSyn PFFs, which reportedly induce less severe and widespread pSer129 aSyn pathology following injections in the rat olfactory bulb compared to mouse PFFs [52, 54]; if this is applicable in our *in vivo* PFF model remains to be addressed. Whether aSyn aggregation and seeding are primary or secondary events in the human MSA disease process and the exact role of p25α in the cascade of events in the context of the human disease are issues that have not yet been elucidated.

In agreement with our *in vivo* findings, the uptake of haSyn PFFs or GCI-related aSyn derived from MSA patients by oligodendrocytes have been reported [50, 55]. However, other studies have failed to detect exogenous haSyn within oligodendrocytes or other glial cells following inoculation with haSyn PFFs [42, 54]. These contradictory findings may be due to differences in the post-injection time frames when the assessments were performed, since although the uptake of injected aSyn by neurons reportedly occurs within minutes, the intraneuronal signal of internalized proteins begins to decrease at 3 h post-injection, probably due to clearance mechanisms [53]. Such a scenario may also apply to oligodendrocytes, given that only one *in vivo* study has shown the presence of exogenous aSyn within oligodendrocytes, assessed at 1 h post-injection [55]. Our *in vitro* data also demonstrate a decrease of the human protein over time, which however may be attributed to clearance mechanisms or to its dilution due to cell proliferation. Another explanation may be that the oligodendroglial uptake of haSyn in the PFF-inoculation models is restricted to near the site of injection, as we have also observed in our model (at least at 1 month post-injection).

Collectively, our study reveals that the administration of haSyn fibrils to cellular systems, i.e. immortalized oligodendroglial cells and differentiated primary oligodendrocytes, recapitulated critical aspects of MSA pathogenesis, thus representing an attractive model system to study the early events leading to GCI formation. Herein, we propose that endogenous aSyn and oligodendroglial phosphoprotein p25α form a dangerous dynamic duo that predisposes oligodendrocytes to accumulate intracellular aSyn aggregates reminiscent of the oligodendroglial inclusions in MSA. Finally, the identification of endogenous oligodendroglial aSyn as a major culprit for the development of pathology *in vitro* and *in vivo*, suggests that manipulation of the expression of aSyn in oligodendrocytes, may provide a rationale approach to combat its accumulation in GCIs and the progression of MSA. An alternative strategy may involve lowering p25α levels, as these also correlate with MSA-type pathology.

## Supplementary Material

1594561_Sup_1

1594561_Sup_2

1594561_Sup_3

1594561_Sup_4

1594561_Sup_5

1594561_Sup_7

1594561_Sup_8

1594561_Sup_9

1594561_Sup_10

1594561_Sup_11

1594561_Sup_12

1594561_Sup_13

1594561_Sup_6
