# Supplementary material for: Endogenous Oligodendroglial Alpha-Synuclein and TPPP/p25α Orchestrate Alpha-Synuclein Pathology in Experimental Multiple System Atrophy Models
Source: Acta Neuropathol. Author manuscript; Available in PMC 2020 Sep 1. (PMC7289399; doi:10.1007/s00401-019-02014-y)
Supplement: 1594561_Sup_1 [file NIHMS1594561-supplement-1594561_Sup_1.docx]

**Electronic Supplementary Material for**

**Endogenous Oligodendroglial Alpha-Synuclein and TPPP/p25α Orchestrate Alpha-Synuclein Pathology in Experimental Multiple System Atrophy Models**

Panagiota Mavroeidi^1^, Fedra Arvanitaki^1^, Anastasia-Kiriaki Karakitsou^1^, Maria Vetsi^1^, Ismini Kloukina^2^, Markus Zweckstetter^3,4^, Karin Giller^4^, Stefan Becker^4^, Zachary A. Sorrentino^5,6^, Benoit I. Giasson^5,6,7^, Poul Henning Jensen^8^, Leonidas Stefanis^1,9^ and Maria Xilouri^1 #^

*^1^Center of Clinical Research, Experimental Surgery and Translational Research, Biomedical Research Foundation of the Academy of Athens, Greece*

*^2^Center of Basic Research, Biomedical Research Foundation of the Academy of Athens, Greece*

*^3^German Center for Neurodegenerative Diseases (DZNE), Von-Siebold-Str. 3a, 37075 Göttingen, Germany*

*^4^Department for NMR-based Structural Biology, Max Planck Institute for Biophysical Chemistry, Am Faßberg 11, 37077 Göttingen, Germany.*

*^5^Department of Neuroscience, University of Florida, Gainesville, FL 32610, USA.*

*^6^Center for Translational Research in Neurodegenerative Disease, University of Florida, Gainesville, FL 32610, USA*

*^7^McKnight Brain Institute, University of Florida, Gainesville, FL 32610, USA*

*^8^DANDRITE-Danish Research Institute of Translational Neuroscience & Department of Biomedicine, University of Aarhus, Denmark*

*^9^1st Department of Neurology, Eginition Hospital, National and Kapodistrian University of Athens, Medical School, Greece*

#Corresponding author

Center of Clinical Research, Experimental Surgery and Translational Research, Biomedical Research Foundation of the Academy of Athens (BRFAA), 4 Soranou Efesiou Street, Athens 11527, Greece

Tel: +30 2106597498; Fax: +30 2106597545;E-mail: [mxilouri@bioacademy.gr](mailto:mxilouri@bioacademy.gr)

**Supplemental Figure Legends**

**Online Resource 1** Human aSyn PFFs are taken up by oligodendroglial cell lines and colocalize with endogenous rat aSyn. **a** OLN-AS7 and OLN-p25α cells overexpress human aSyn and p25α proteins, respectively. Representative immunoblots for p25α, haSyn (human-specific antibody 4B12), total aSyn (C20 antibody, recognizes endogenous and human aSyn), and β-actin as a loading control in the Triton-soluble fraction. **b** (upper rows) Representative immunofluorescence images of OLN-93 cells transfected with haSyn or p25α plasmids for 48 h using antibodies against haSyn (green, LB-509 antibody, upper panel) or p25α (red, bottom panel). (bottom row) Quantification of cell viability following transfection of all OLN cell lines with GFP, haSyn of p25α plasmids for 48 h. Data are expressed as the mean ± SE of at least three independent experiments with triplicate samples/condition within each experiment. **c** Transmission EM image of negative stained haSyn PFF samples confirming the presence of fibrils. Scale bar: 500 nm. **d** Biochemical profile of aSyn species formed in OLN-93 cells following the addition of 0.2 and 0.5 μg recombinant haSyn PFFs or PBS as a control at 48 h post-addition. Representative immunoblots for both monomeric and HMW species of aSyn (human-specific antibody 4B12) and β-actin as a loading control in Triton-, SDS-, and urea-soluble fractions. Asterisk represents non-specific bands obtained with the human-specific 4B12 antibody (detected also in PBS-treated OLN-93 cells in which no haSyn was present). **e** aSyn aggregates, composed of human and endogenous rat aSyn, are located in the perinuclear space and cytoplasm of OLN-93 cells following the addition of 0.5 μg haSyn PFFs for 48 h. Representative immunofluorescence images with antibodies against endogenous rat aSyn (green, D37A6 antibody) and haSyn (gray, LB-509). Wheat germ agglutinin was used as a plasma membrane marker (red, WGA) and DAPI as a nuclear marker. Scale bar: 10 μm.

**Online Resource 2** Quantification of relative human and total aSyn levels (monomeric and HMW species) in the Triton- and SDS-soluble fraction of OLN cells treated with 0.5 μg haSyn PFFs. **a, b** Quantification of monomeric human (left panels) and total (right panels) aSyn levels detected in the Triton-soluble fraction of OLN-93 and OLN-AS7 cells (**a**) or OLN-93 and OLN-p25α cells (**b**) treated with 0.5 μg haSyn PFFs (or PBS as a control) for 24-96 h. **c-f** Quantification of monomeric (upper panels) and HMW species (bottom panels) of human (left) and total (right) aSyn protein levels detected in the SDS-soluble fraction of OLN-93 and OLN-AS7 cells (**c** and **e**) or OLN-93 and OLN-p25α cells (**d** and **f**) treated with 0.5 μg haSyn PFFs (or PBS as a control) for 24-96 h. Data are expressed as the mean ± SE of five independent experiments with triplicate samples/condition within each experiment; *p < 0.05; **p < 0.01; ***p < 0.001, by two-way ANOVA with Bonferroni’s correction, comparing between OLN-93 and OLN-AS7 or OLN-p25α cells treated with haSyn PFFs at all time points.

**Online Resource 3** High-power images demonstrating the recruitment of endogenous rat aSyn in all OLN cell lines following the addition of 0.5 μg of haSyn PFFs for 48 h and 10 days using antibodies against haSyn (green, LB-509 antibody) and endogenous rat aSyn (red, D37A6 antibody) and DAPI staining. Scale bar: 10 μm.

**Online Resource 4** Co-localization of exogenously added haSyn with endogenous rat aSyn following the addition of increasing amounts of haSyn PFFs to OLN-93 cells. The endogenous rat aSyn signal is increased following the incubation of OLN-93 cells even with small amounts of haSyn PFFs (0.1 μg) for 48 h. Representative immunofluorescence images with antibodies against haSyn (green, LB-509 antibody) and endogenous rat aSyn (red) and DAPI staining. Scale bar: 25 μm.

**Online Resource 5** Stable overexpression of p25α promotes the formation of proteinase K-resistant aSyn inclusions formed following the addition of haSyn PFFs. **a** Representative immunofluorescence images using antibodies against haSyn (green, LB-509 antibody) and endogenous rat aSyn (red, D37A6 antibody) and DAPI staining in OLN cell lines treated with 0.05 μg/mL proteinase K for 15 min (PFF+ProtK) following the addition of 0.5 μg haSyn PFFs for 48 h. Scale bar: 25 μm. **b** Quantification of human (left) and endogenous rat (right) aSyn levels measured as % area surface/cell in PFF-treated (0.5 μg for 48 h) OLN cell lines without (PFFs) or following proteinase-K (PFF+ProtK) treatment. Data are expressed as the mean ± SE of three independent experiments with triplicate samples/condition within each experiment. *p < 0.05; **p < 0.01; ***p < 0.001 by two-way ANOVA with Bonferroni’s correction.

**Online Resource 6** Specificity of the rodent-specific aSyn antibody D37A6. **a** Human SHSY5Y cells inducibly over-expressing human aSyn were treated with 0.5 μg haSyn PFFs for 48h. No signal from the rodent-specific aSyn D37A6 antibody was detected. Representative immunofluorescence images with antibodies against haSyn (green, LB-509 antibody) and rodent aSyn (red) and DAPI staining. Scale bar: 25 μm. **b** (Upper panel) Representative immunoblots for rodent-specific aSyn and actin in: total brain lysates derived from mice expressing endogenous WT protein (WT-aSyn), mice overexpressing hA53T-aSyn in neurons (hA53T-aSyn), aSyn KO mice (KO-aSyn), SDS-soluble fraction of SH-SY5Y cells pre-treated with haSyn PFFs, and Triton-, SDS-, and urea-soluble fractions of rat OLN-93 cells treated with haSyn PFFs for 96 h. (Lower panel) Representative immunoblots for rodent aSyn (D37A6 antibody, left) and total aSyn (C20 antibody, right) in WT-aSyn mouse brain lysate run together with 0.1 μg haSyn PFFs, further confirming the specificity of the D37A6 aSyn antibody to recognize only endogenous rodent aSyn and not exogenously added human fibrils. **c** Endogenous rat aSyn accumulates following treatment of OLN-93 cells with proteasomal (epoxomicin, 15 nM) or total lysosomal (NH_4_Cl, 20 mM) inhibitors for 48 h, alone or in combination. Representative immunofluorescence images with antibodies against rodent aSyn (D37A6 antibody, red) and α-tubulin (green) and DAPI staining. Scale bar: 10 μm. **d** Representative immunoblots for rodent aSyn (D37A6 antibody), poly-ubiquitinated proteins, LC3I, LC3II, and p62 (as autophagy markers), and β-actin (as a loading control), verifying the presence of endogenous oligodendroglial aSyn following treatment of OLN-93 cells with epoxomicin or/and NH_4_Cl for 48 h.

**Online Resource 7** Addition of haSyn PFFs to OLN cells is accompanied by the formation of intracellular insoluble aSyn aggregates, which remain in a fibrillar state even following prolonged incubation with PFFs. **a** Representative immunofluorescence images depicting the aSyn aggregates in OLN-93, OLN-AS7, and OLN-p25α cells following the addition of 0.5 μg haSyn PFFs at 1 h, 48 h and 10 days using antibodies against total (human+rodent) aSyn (green, D10 antibody) and aggregated aSyn (red, MJFR-14 antibody) and DAPI staining. Scale bar: 25 μm. **b** Incubation of OLN cells with 3 μg haSyn PFFs for 48 h or 10 days expedites the formation of highly aggregated aSyn forms. Representative immunofluorescence images with antibodies against total aSyn (green, D10 antibody) and aggregated aSyn (red, MJFR-14) and DAPI staining. Scale bar: 25 μm. **c** (upper panels) Representative immunofluorescence images with antibodies against human aSyn (green, 211 antibody) and aggregated aSyn (red, MJFR-14) and DAPI staining. Scale bar: 25 μm. (bottom panel) Quantification of total (left) and aggregated (right) aSyn levels measured as % area surface/cell in OLN cell lines following the addition of 3 μg haSyn PFFs for 48 h or 10 days. Data are expressed as the mean ± SE of three independent experiments with triplicate samples/condition within each experiment. *p < 0.05; **p < 0.01 by two-way ANOVA with Bonferroni’s correction.

**Online Resource 8** The addition of sonicated haSyn PFFs leads to the recruitment of endogenous rat aSyn and the formation of pathological aSyn species. **a-c** Representative immunofluorescence images of all OLN cell lines treated with non-sonicated and sonicated PFFs (0.5 μg for 48 h) using antibodies against human aSyn (green, LB-509 antibody), endogenous rat aSyn (red, D37A6 antibody), oxidized/nitrated aSyn (green, Syn303 antibody) and aggregated aSyn (red, MJFR-14 antibody) and DAPI staining. Scale bar: 25 μm. **d** Transmission EM images of negatively stained haSyn PFF samples pre-sonication (upper) or post-sonication (bottom) confirming the efficient sonication of the fibrils utilized in **a-c**.

**Online Resource 9** Unilateral intrastriatal delivery of PBS into the mouse WT-aSyn brain is not associated with aSyn-related pathology. **a–d** Representative immunofluorescence images with antibodies against haSyn (gray, LB-509 antibody), total aSyn (gray, SYN1 antibody), oxidized/nitrated aSyn (gray, Syn303 antibody), endogenous mouse aSyn (green, D37A6 antibody), aggregated aSyn (green, MJFR-14 antibody), p25α (red, shown in **d**), and MBP (red, shown in **a–c**) and DAPI staining. Scale bar: 10 μm.

**Supplemental Tables**

**Online Resource 10** Details of the post-mortem autopsy cases used in this study. MSA-P, multiple system atrophy-parkinsonism; MSA-C, multiple system atrophy-cerebellar; DLB, dementia with Lewy bodies; AD, Alzheimer’s disease; CAA, cerebral amyloid angiopathy. The Thal phasing scale and the Consortium to Establish a Registry for Alzheimer’s disease (CERAD) neuropathological criteria were utilized to measure neuritic plaques and Braak stage for neurofibrillary tangles.

**Online Resource 11** A table depicting the primary and secondary antibodies used either in western blotting or immunocytochemistry/immunohistochemistry including species reactivity and working dilutions.

**Online Resource 12** A data summary table of mean values ± SD from statistical analysis of all quantitative data provided in Figs. 1–5 and 7–9 and ESM_Figs. 1, 2, 5, and 7.

**Supplemental Videos**

**Online Resource 13** Three-dimensional immunocytochemical visualization of haSyn PFFs within the boundaries of OLN-93 cells. OLN-93 cells were treated with 0.5 μg haSyn PFFs for 48 h, followed by fixation and staining with an haSyn antibody (gray, LB-509 antibody) and wheat germ agglutinin (red, WGA) as a plasma membrane marker. Stained cells were imaged using a 63× oil immersion objective (depth = 13.1 μm, step size = 0.5 μm). The 3-D image was analyzed by Volocity Image Analysis Software (AVI 11,100 kb).

**Online Resource 14** Three-dimensional immunohistological visualization of recruited endogenous rodent aSyn co-localized with exogenously added haSyn PFFs in MBP^+^ cells in the ipsilateral (IPSI) striatum. HaSyn PFFs (4 μg) were delivered into the WT-aSyn mouse striatum. At 1 month post-injection, the mice were sacrificed and brain sections were stained with antibodies against haSyn (gray, LB-509 antibody), endogenous rodent aSyn (green, D37A6 antibody), and MBP (red). The stained tissue was imaged using a 63× oil immersion objective (depth = 23.2 μm, step size = 0.5 μm). The 3-D image was analyzed by Volocity Image Analysis Software (AVI 138,000 kb).

**Online Resource 15** Three-dimensional immunohistological visualization of haSyn PFFs within p25α^+^ oligodendrocytes in the injected mouse striatum. At 1 month post-injection of 4 μg haSyn PFFs into the WT-aSyn mouse striatum, brain sections were stained with antibodies against haSyn (green, LB-509 antibody) and p25α (red). The stained tissue was imaged using a 63× oil immersion objective (depth = 8.1 μm, step size = 1.01 μm). The 3-D image was analyzed by Volocity Image Analysis Software (AVI 16,000 kb).

**Supplemental Methods (Online Resource 16)**

**Cell culture and transfections of OLN cell lines**

For the immunofluorescence experiments, the cells were seeded on poly-D-lysine-coated coverslips (P7405; Sigma-Aldrich, St. Louis, MO, USA) in 24-well plates at a density of 10,000 cells/well (for analysis up to 48 h) or 1,000 cells/well (for 10 days). For the biochemical analysis, the cells were plated on 6-well plates at a density of 200,000 cells/well (for treatment up to 96 h) or 50,000 cells/well (for treatment up to 10 days). PFFs were added the following day at a final concentration of 0.5 μg/mL culture medium/well (0.5 µg PFFs) or 3 μg/mL culture medium/well (3 μg PFFs). Transient transfections with haSyn, p25α, and/or GFP plasmids were performed with polyethylenimine-PEI (26966; Polysciences, Germany) reagent, and the cells were fixed at the indicated time points using 4% paraformaldehyde (P6148; Sigma-Aldrich, St. Louis, MO, USA) in PBS and processed for immunofluorescence analysis. To stain the lectins of the plasma membrane, wheat germ agglutinin (Alexa Fluor™ 488-conjugated; W11261; Thermo Fisher Scientific, Waltham, MA, USA) was added to PFF-treated OLN-93 cells (0.5 μg, 48 h) for 10 min at 37°C (concentration, 1 μg/mL), prior to fixation for confocal microscopy analysis. For proteinase K treatment, OLN cells were treated with 0.05 μg/mL proteinase-K (AM2542; Thermo Fisher Scientific, Waltham, MA, USA) for 15 min following the addition of 0.5 μg haSyn PFFs for 48 h. To inhibit cellular protein catabolism, OLN-93 cells were treated with the proteasomal inhibitor epoxomicin (15 nM) or the general lysosomal inhibitor NH_4_Cl (20 mM) for 48 h, and the cells were processed for immunofluorescence analysis as above.

**Primary oligodendroglial cultures**

Briefly, mixed glial cultures were maintained in Dulbecco’s modified Eagle’s medium supplemented with 10% fetal bovine serum and 1% penicillin/streptomycin for 10–14 days. Loosely attached microglia were removed by shaking for 1 h (200 rpm, 37°C), followed by additional shaking at 200 rpm for 18 h at 37°C for the enrichment of the culture with OPCs. The cells were then seeded at a density of 80,000 cells/well on poly-D-lysine-coated coverslips in 24-well plates and cultured in SATO medium [[1](#_ENREF_1)] supplemented with insulin-transferrin-selenium solution (41400045; Gibco, Carlsbad, CA, USA), 1% penicillin/streptomycin, and 1% horse serum (H1138; Sigma-Aldrich, St. Louis, MO, USA). The cells were grown in SATO medium for 4 days prior to the addition of 0.5 µg haSyn PFFs (final concentration: 0.5 µg/mL culture medium/well). Differentiation of OPCs to mature oligodendrocytes was verified by labeling the cultures with an anti-MBP antibody (1:200; MCA409S; Bio-Rad, Hercules, CA, USA).

**Preparation of primary cortical neuron cultures**

Dissociated cells were plated onto poly-D-lysine-coated 24-well dishes at a density of 100,000–120,000 cells/well. The cells were grown in Neurobasal/B27 medium for 5 days prior to the addition of 3 μg haSyn PFFs (final concentration: 3 μg/mL culture medium/well of a 24-well plate).

**Subcellular fractionation and western immunoblotting**

The cells were solubilized in a 1% Triton X-100-containing buffer (150 mM NaCl, 50 mM Tris pH 7.6, 2 mM EDTA), left on ice for ~30 min, and centrifuged at 10,000 × *g* for 15 min at 4°C to obtain the Triton-soluble fraction. The pellet was washed twice with PBS and solubilized in 1% SDS-containing buffer (150 mM NaCl, 50 mM Tris pH 7.6, 2 mM EDTA), sonicated, and centrifuged as above to obtain the SDS-soluble fraction. The resulting pellet, after two washes with 5% SDS, was solubilized in 8 M urea-5% SDS-containing buffer, heated for 30 min at 45°C, and centrifuged as above to generate the urea-soluble fraction. All buffers were supplemented with protease (11836170001; Roche, Mannheim, Germany) and phosphatase inhibitors (04406837001; Roche, Mannheim, Germany).

**Assessment of survival**

The cells were transfected with haSyn, p25α, or GFP plasmids for 48 h and lysed in a detergent-containing solution. Cell survival was assessed by counting the number of intact nuclei in a hemocytometer as described previously [[2](#_ENREF_2), [4](#_ENREF_4)]. Cell counts were performed in triplicate and are reported as means ± SE.

**Sonication of haSyn PFFs**

Human aSyn PFFs were sonicated using a probe tip sonicator as described previously [[6](#_ENREF_6)]. Briefly, a working solution of haSyn PFFs was prepared at a final concentration of 0.1 mg/mL and sonicated with 60 pulses at 10% power (total of 30 s, 0.5 s on, 0.5 s off). The cells were incubated with 0.5 μg haSyn PFFs (pre- and post-sonication) or PBS as a control for 48 h and processed for confocal microscopy.

**Transmission EM**

***Preparation of cultured cells for EM and immuno-EM*:** For conventional EM, the cells were fixed for 1 h in 2.5% glutaraldehyde (at 37°C) made up in 0.1M phosphate buffer (pH 7.4). After subsequent buffer washes, the cells were released from the dish by gentle scraping and pelleted at 800 x *g* for 5 min. The pellets were embedded in 4% low-melting-point agarose in 0.1 M phosphate buffer. After solidification, small cubes were cut and post-fixed with 1% osmium tetroxide for 1 h on ice. After washing with the above buffer, the samples were dehydrated in a graded ethanol series and embedded in an Epon/Araldite resin mixture. Ultrathin sections were cut with a DiATOME diamond knife at a thickness of 65 nm on a Leica EM UC7 ultramicrotome (Leica Microsystems, Vienna, Austria), mounted onto 300 mesh copper grids, and stained with uranyl acetate and lead citrate. For immuno-EM, the cells were fixed for 1 h in 3% paraformaldehyde/0.5% glutaraldehyde made up in 0.1 M phosphate buffer and processed as above. Post-embedding double immunogold labeling was performed on thin sections for the detection of human and rodent aSyn. Briefly, the grids were floated on a blocking solution (0.05 M Tris-HCl, pH 7.4, 1% bovine serum albumin [BSA], 0.1% cold water fish gelatin, 5% normal goat serum, 0.1% Tween 20) for 30 min. The grids were then incubated in a mixture of the LB-509 mouse monoclonal antibody (1:100) and D37A6 rabbit monoclonal antibody (1:100) diluted in Tris-HCl/BSA, overnight at 4°C. After several washes with Tris-HCl/BSA, the grids were incubated for 1 h at room temperature in a mixture of goat anti-mouse IgG conjugated to 10-nm gold particles [1:40] and goat anti-rabbit IgG conjugated to 15-nm gold particles [1:40]. Finally, the grids were counterstained with ethanolic uranyl acetate and lead citrate.

***EM for myelin integrity:*** At 1 month post-injection, haSyn PFF-treated WT- and KO-aSyn mice (n = 4/genotype) were anesthetized and perfused transcardially with 0.1 M PBS (pH 7.2) at 37°C, with a flow rate of 10 mL/min for 5 min and then with a 4% paraformaldehyde/1% glutaraldehyde fixative mixture at the same rate for 15 min. The brain was removed and the ipsilateral striatum was dissected into small pieces and placed in fixative for 1 h at 4°C. After subsequent buffer washes, the tissue was cut into small pieces and post-fixed in 1% osmium tetroxide/1.5% potassium ferrocyanide for 1 h on ice to preserve myelin integrity further [[4](#_ENREF_4)]. This was followed by immersion of the blocks in 2% glutaraldehyde for 1.5 h on ice. After washing, the samples were dehydrated and embedded in an Epon/Araldite resin mixture. Ultrathin sections (65 nm) were cut, mounted onto 300 mesh copper grids, and stained with uranyl acetate and lead citrate as above.

**RNA Extraction, cDNA synthesis, and real-time PCR**

Duplicates of each sample were assayed by relative quantitative real-time PCR using a Light Cycler 96 (Roche Applied Science, Mannheim, Germany) machine to determine the mRNA expression levels of rat *Snca*. As a reference gene for normalization, we used β-actin. The primers used for rat *Snca* were 5′-GCCTTTCACCCCTCTTGCAT-3′ (forward), 5′-TATCTTTGCTCCACACGGCT-3′ (reverse) and for β-actin, 5′-TGGCTCCTAGCACCATGA-3′ (forward), 5′-CCACCAATCCACACAGAG-3′ (reverse). Each cDNA sample was diluted 1:20 before use in the amplification assay. The PCR conditions were as follows: 1× buffer (-Mg), 1.5 mm MgCl_2_, 0.2 mm dNTPs, 0.2 μm primers, template < 500 ng, 2 U Platinum *Taq*, and SYBR Green (Roche, Mannheim, Germany). The PCR cycling conditions were as follows: 95°C for 180 s, 95°C for 10 s, 60°C for 15 s, and 72°C for 15 s (45 cycles), and 95°C for 60 s, 65°C for 60 s, 95°C for 10 s, and 37°C for 30 s. As a negative control for the specificity of amplification, we used no template samples in each plate; no amplification product was detected in the control reactions. Data were analyzed automatically with a threshold set in the linear range of amplification. The cycle number at which any particular sample crossed that threshold (*Ct*) was used to determine fold difference, whereas the geometric mean of the control gene (β-actin) served as a reference for normalization. Fold difference was calculated with the 2−ΔΔ*Ct* method [[3](#_ENREF_3)].

**References**

1 Bottenstein JE, Sato GH (1979) Growth of a rat neuroblastoma cell line in serum-free supplemented medium. Proc Natl Acad Sci U S A 76: 514-517

2 Farinelli SE, Greene LA, Friedman WJ (1998) Neuroprotective actions of dipyridamole on cultured CNS neurons. J Neurosci 18: 5112-5123

3 Livak KJ, Schmittgen TD (2001) Analysis of relative gene expression data using real-time quantitative PCR and the 2(-Delta Delta C(T)) Method. Methods 25: 402-408 Doi 10.1006/meth.2001.1262

4 MJ Hooshmand AA, BJ Cummings (2014) Improved pre-embedded immuno-electron microscopy procedures to preserve myelin integrity in mammalian central nervous tissue. Microscopy: advances in scientific research and education 6: 59-65

5 Rukenstein A, Rydel RE, Greene LA (1991) Multiple agents rescue PC12 cells from serum-free cell death by translation- and transcription-independent mechanisms. J Neurosci 11: 2552-2563

6 Volpicelli-Daley LA, Luk KC, Lee VM (2014) Addition of exogenous alpha-synuclein preformed fibrils to primary neuronal cultures to seed recruitment of endogenous alpha-synuclein to Lewy body and Lewy neurite-like aggregates. Nat Protoc 9: 2135-2146 Doi 10.1038/nprot.2014.143
