# Supplementary figures and images for: Endogenous Oligodendroglial Alpha-Synuclein and TPPP/p25α Orchestrate Alpha-Synuclein Pathology in Experimental Multiple System Atrophy Models
Source: Acta Neuropathol. Author manuscript; Available in PMC 2020 Sep 1. (PMC7289399; doi:10.1007/s00401-019-02014-y)

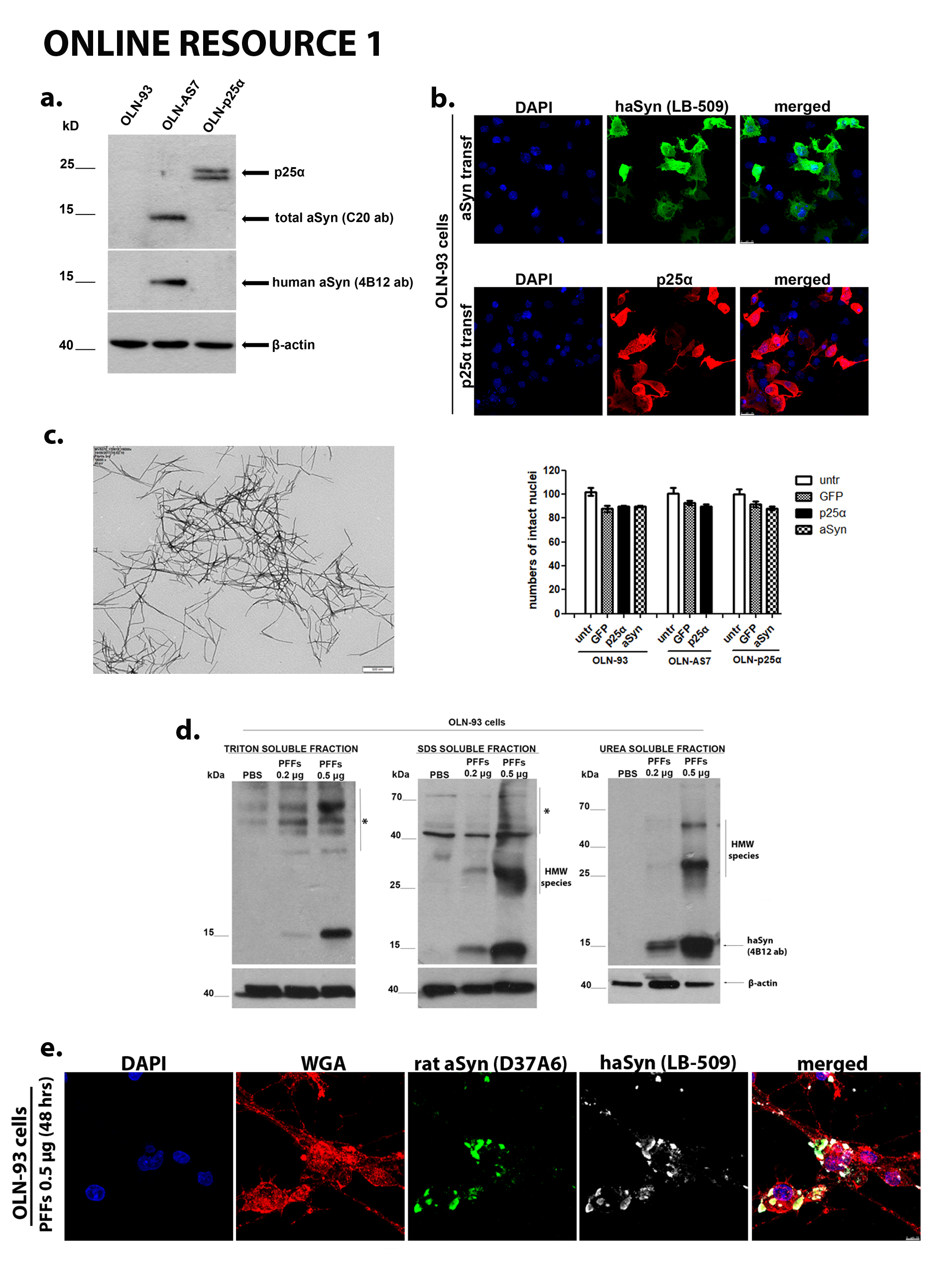

Supplement: 1594561_Sup_2 [file NIHMS1594561-supplement-1594561_Sup_2.tif]

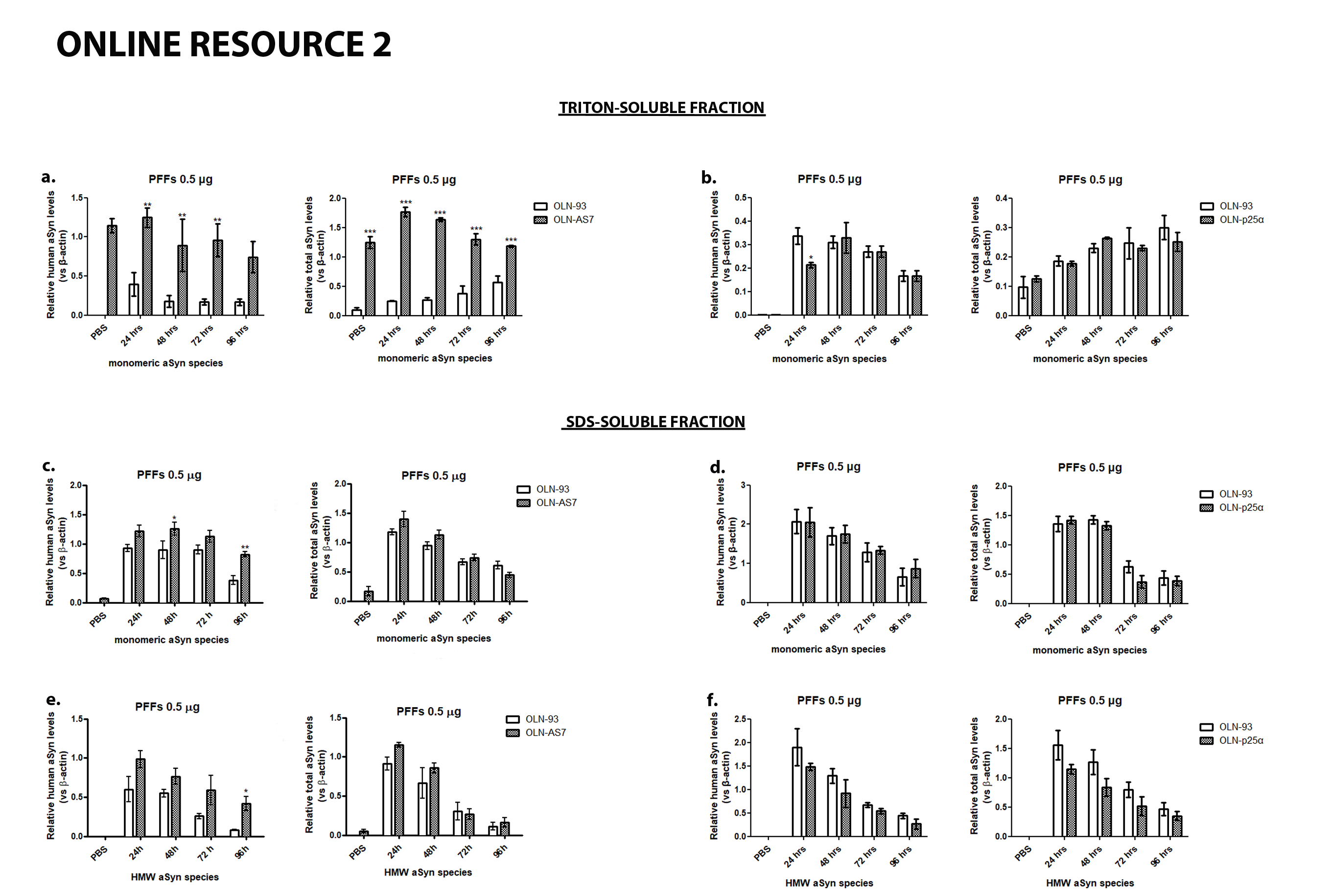

Supplement: 1594561_Sup_3 [file NIHMS1594561-supplement-1594561_Sup_3.tif]

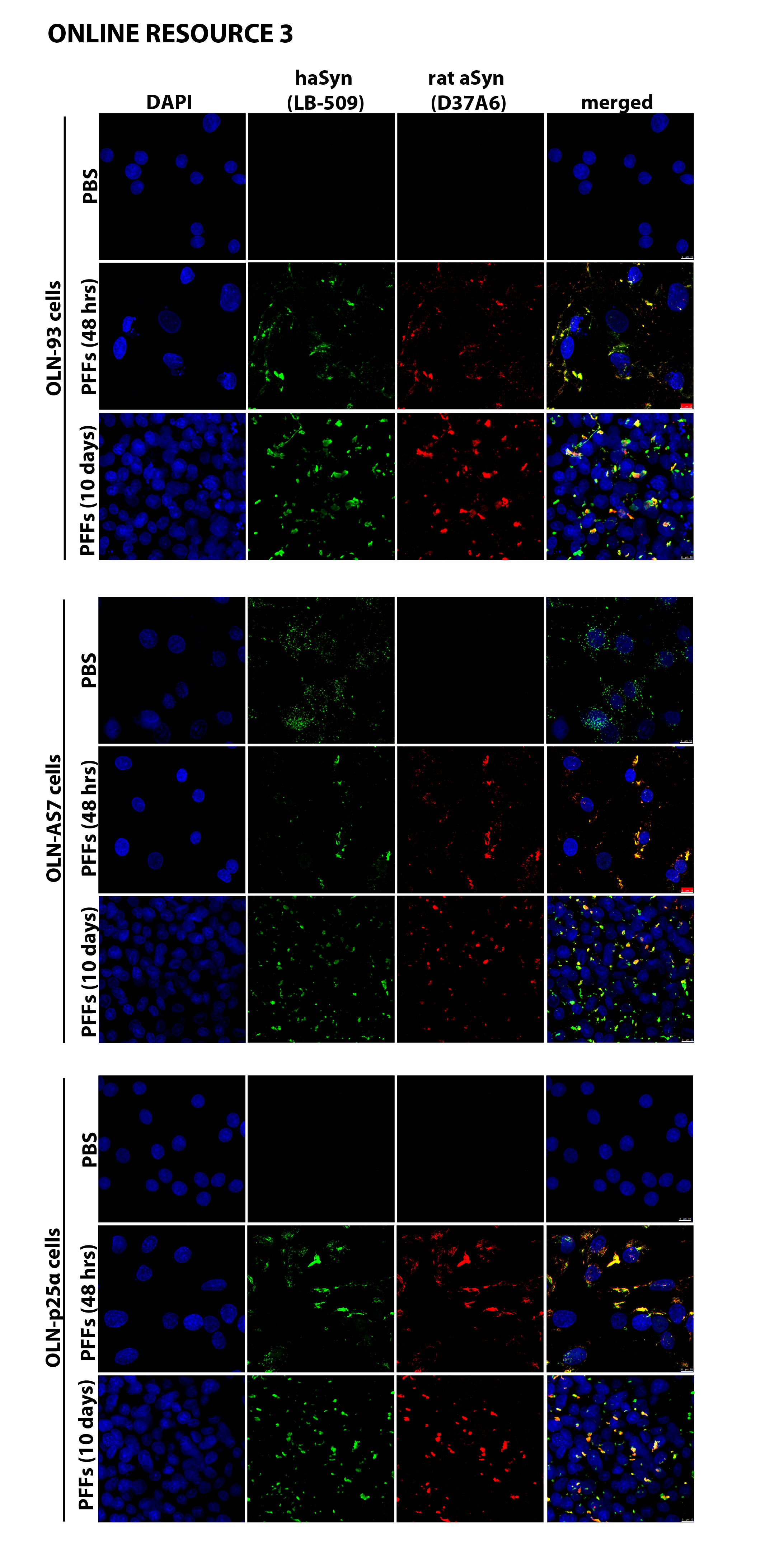

Supplement: 1594561_Sup_4 [file NIHMS1594561-supplement-1594561_Sup_4.tif]

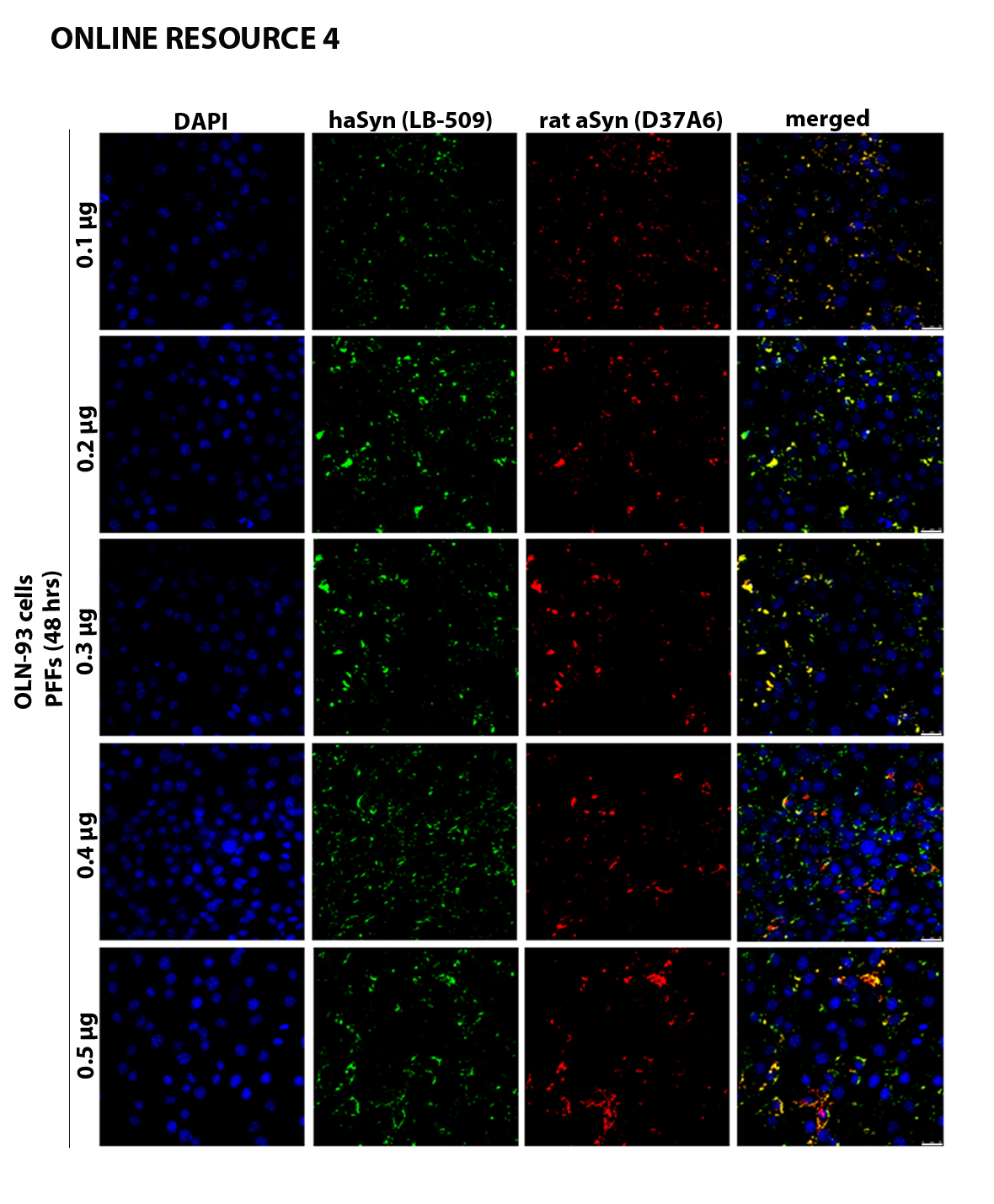

Supplement: 1594561_Sup_5 [file NIHMS1594561-supplement-1594561_Sup_5.tif]

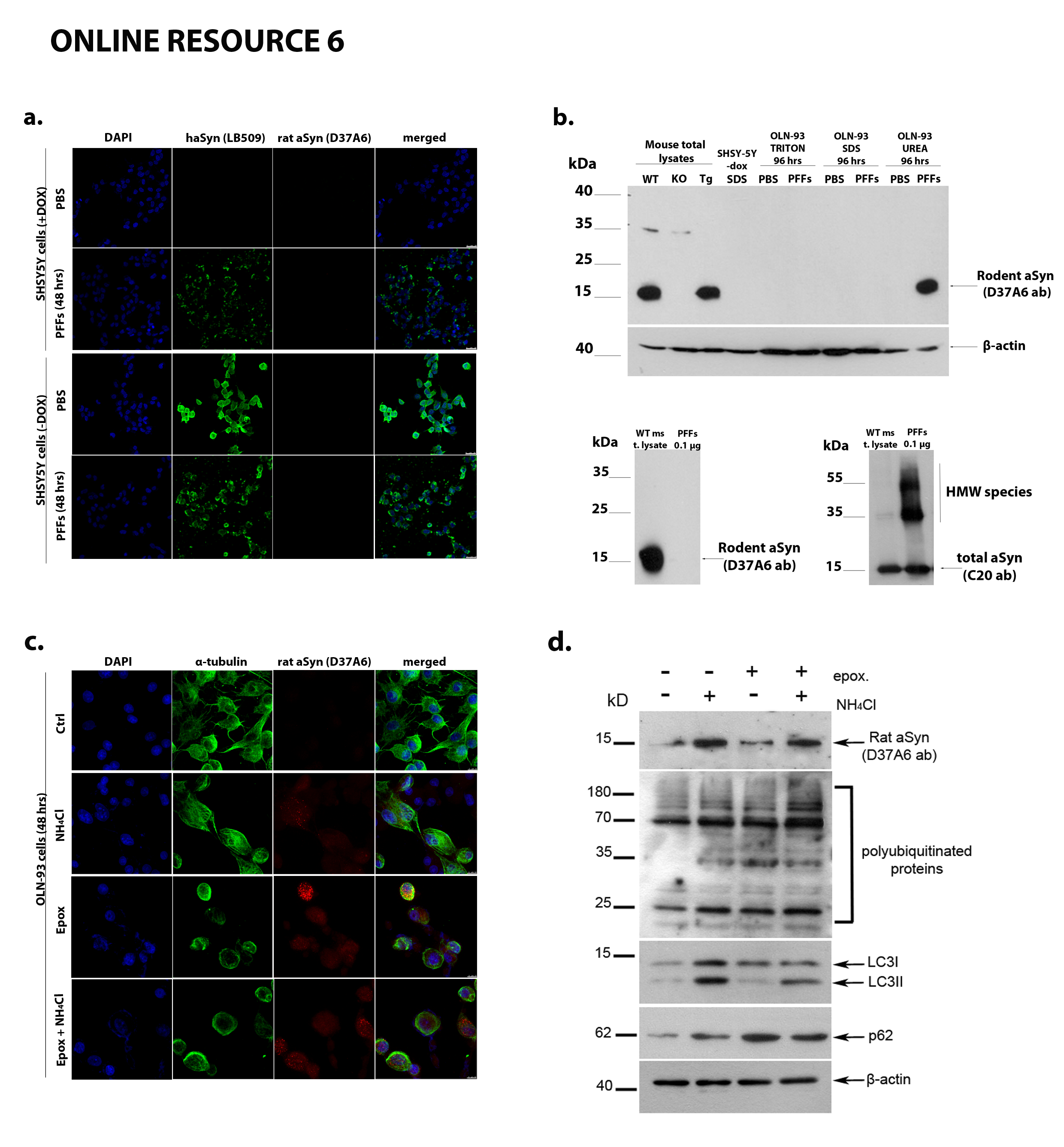

Supplement: 1594561_Sup_7 [file NIHMS1594561-supplement-1594561_Sup_7.tif]

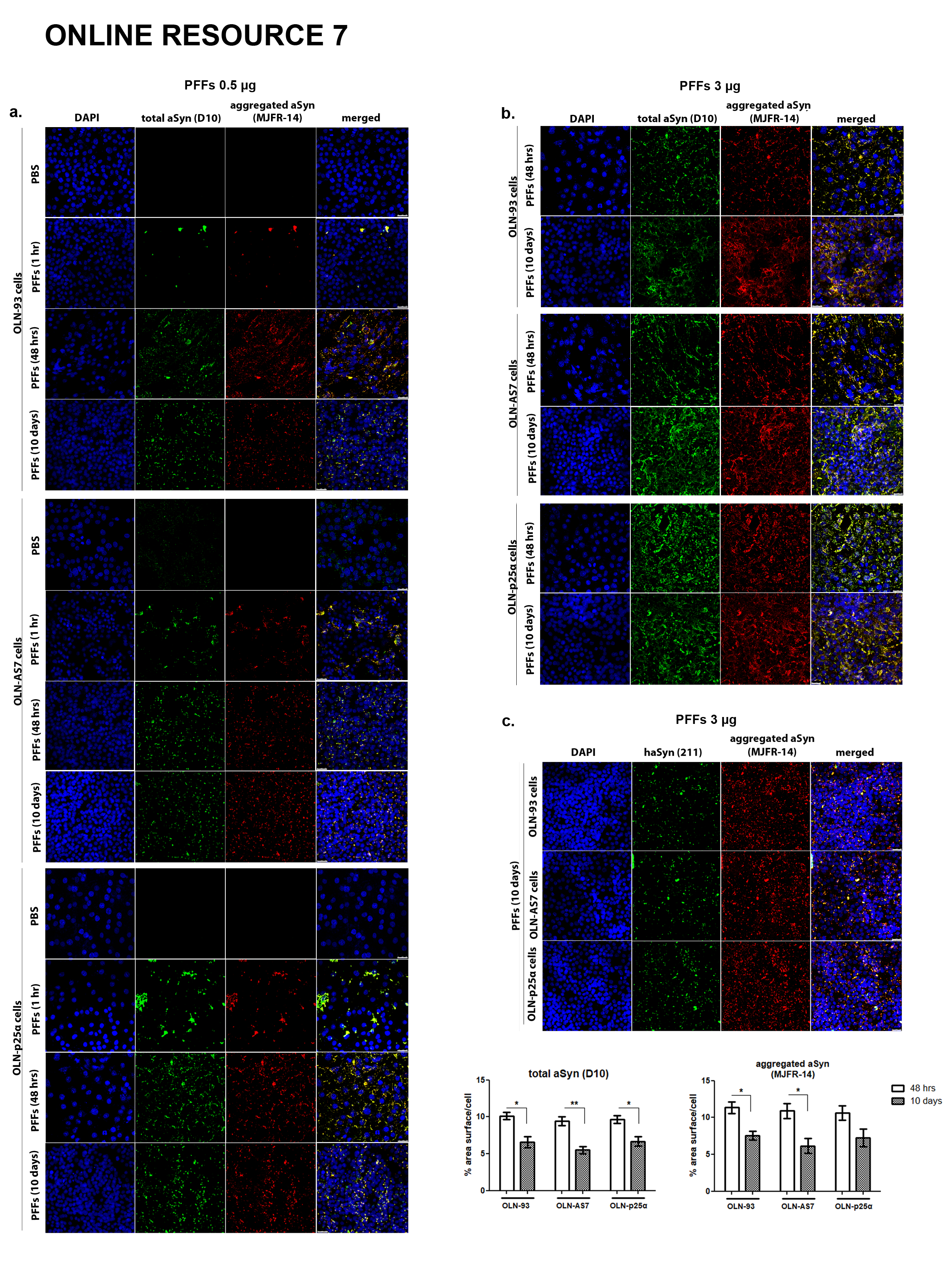

Supplement: 1594561_Sup_8 [file NIHMS1594561-supplement-1594561_Sup_8.tif]

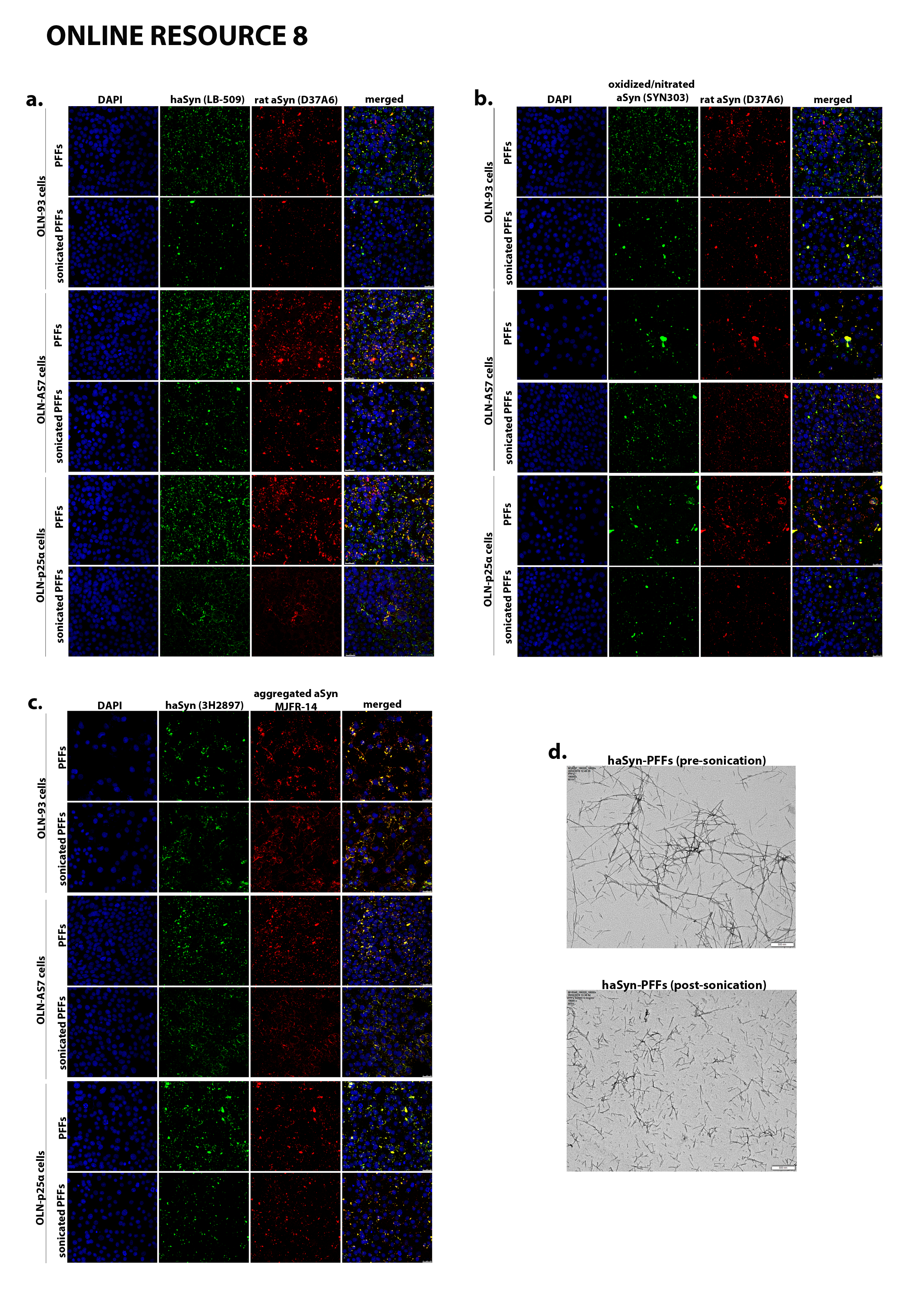

Supplement: 1594561_Sup_9 [file NIHMS1594561-supplement-1594561_Sup_9.tif]

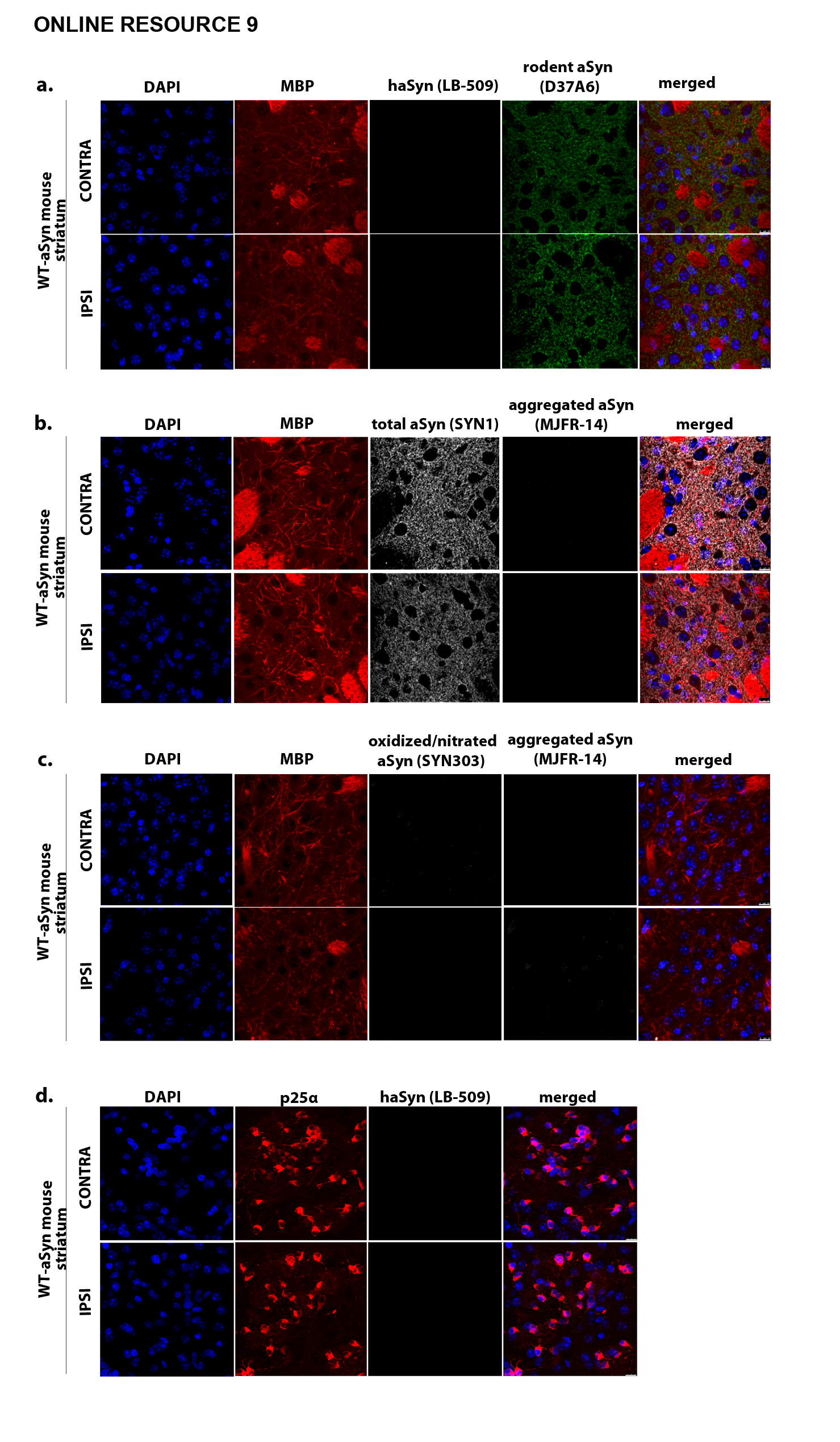

Supplement: 1594561_Sup_10 [file NIHMS1594561-supplement-1594561_Sup_10.tif]

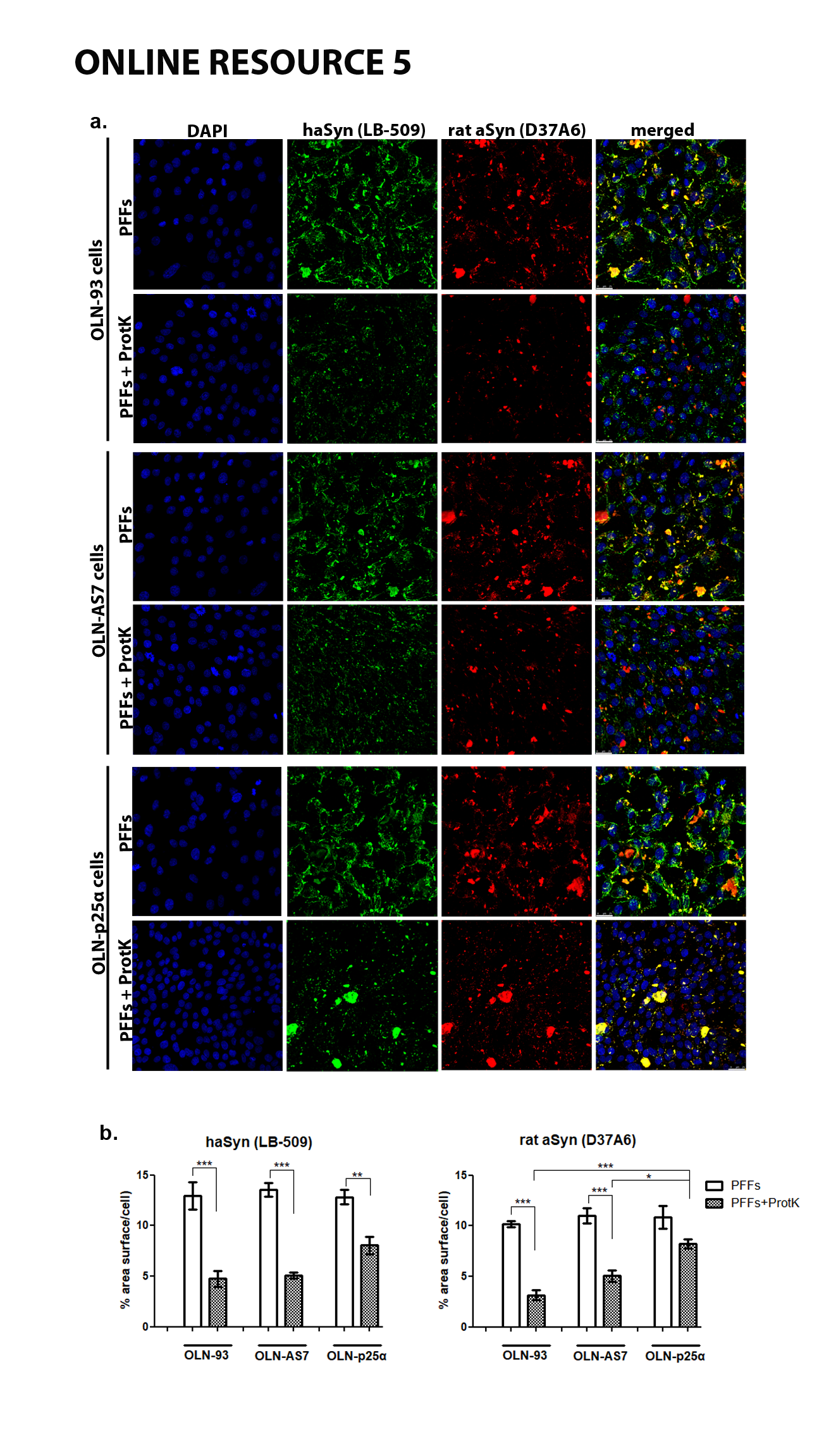

Supplement: 1594561_Sup_6 [file NIHMS1594561-supplement-1594561_Sup_6.tif]
